# Gel Filtration Chromatography-Guided Sequential Hydrolysis of *Sthenoteuthis* *oualaniensis* Protein: Peptide Distribution and Functional Properties

**DOI:** 10.3390/foods15172998

**Published:** 2026-08-26

**Authors:** Qian Yao, Huiying Wang, Haoze Yang, Ruofei Hong, Yong Zhong, Xiaozhen Diao, Wenhui Wu

**Affiliations:** 1 Department of Marine Biopharmacology, College of Food Science and Technology, Shanghai Ocean University, Shanghai 201306, China; 2 Marine Biomedical Science and Technology Innovation Platform of Lin-gang Special Area, Shanghai 201306, China; 3 Putuo Branch of International Combined Research Center for Marine Biological Sciences, Zhoushan 316104, China

**Keywords:** *Sthenoteuthis oualaniensis*, sequential enzymatic hydrolysis, gel filtration chromatography, optimized design, amino acid analysis, degree of hydrolysis, relative radical-scavenging capacity

## Abstract

The compact muscle architecture and poor aqueous dispersibility of *Sthenoteuthis oualaniensis* protein limit its use as a food ingredient. This study developed a gel filtration chromatography (GFC)-guided papain–alcalase sequential hydrolysis strategy using target-window peak area ($A_{\mathrm{target}}$) as a peptide-distribution response. Box–Behnken optimization identified an enzyme dosage of 1110 U/g, a papain/alcalase mass ratio of 3:5, and 8 h hydrolysis, yielding an $A_{\mathrm{target}}$ of 0.0648 ± 0.0012 a.u. O-phthaldialdehyde (OPA)-derived degree of hydrolysis (DH) values were 10.72 ± 0.08%, 35.32 ± 0.12%, and 25.03 ± 0.37% for SPH-Pap, SPH-Alc, and SPH-opt, respectively, showing that the highest $A_{\mathrm{target}}$ did not coincide with the highest DH. The essential-to-total amino acid ratio remained 38.18–39.20%, while lysine and methionine changed modestly. SPH-opt maintained high solubility across a broad pH range and exhibited a peptide profile distinct from those of the single-enzyme hydrolysates. Radical-scavenging capacity was assessed only post-optimization. At 5 mg/mL, SPH-opt showed DPPH and ABTS scavenging rates of 23.75% and 22.84%, respectively; without external standards, these values support only relative within-study comparisons. Overall, $A_{\mathrm{target}}$ and DH provided complementary information on peptide distribution and bond cleavage, and no causal relationship between $A_{\mathrm{target}}$ and radical-scavenging capacity was established.

## 1. Introduction

*Sthenoteuthis oualaniensis*, a squid species widely distributed in the Indian and Pacific Oceans, has been regarded as a promising marine bioresource because of its large biomass, high protein content, and low lipid level [1]. However, the tightly arranged muscle fibers and tough texture of *S. oualaniensis*, together with off-flavor formation caused by endogenous enzymes during storage and processing, have largely confined current utilization to primary products such as frozen squid [2]. Among the available routes for value-added conversion, enzymatic hydrolysis offers a practical means of cleaving peptide bonds in proteins in a site-selective manner, converting macromolecular myofibrillar proteins into lower-molecular-weight peptides with improved solubility and potential functional properties [3,4,5]. A controllable hydrolysis strategy tailored to *S. oualaniensis* protein is therefore needed to improve processing adaptability and broaden its application as a functional food ingredient [6,7].

Most studies on enzymatic hydrolysis of marine proteins have relied on a single protease, such as alcalase or papain [8]. Although single-enzyme hydrolysis is operationally simple, hydrolytic performance is constrained by cleavage-site specificity. As a result, incomplete protein degradation, insufficient release of target peptides, or, under excessive hydrolysis, further conversion into overly small peptides and free amino acids may occur [9,10]. By contrast, combined-enzyme or sequential hydrolysis can exploit the complementary cleavage preferences of different proteases, thereby reshaping peptide composition and functional properties [11]. Papain exhibits preferences for hydrophobic amino acid residues and may facilitate the release of hydrophobic fragments and peptides containing aromatic residues. Alcalase, a broad-specificity serine endopeptidase, can further cleave accessible protein backbones and increase the exposure of accessible cleavage sites [12,13]. Sequential hydrolysis using papain followed by alcalase may therefore create a possible complementary cleavage pattern between initial structure loosening and subsequent backbone cleavage, although this design does not by itself prove that the papain–alcalase order is superior to reverse-order or simultaneous dual-enzyme hydrolysis.

At present, enzymatic hydrolysis processes are usually optimized using the degree of hydrolysis (DH) as the primary evaluation index [14,15]. DH is useful for describing the overall extent of peptide-bond cleavage and remains a basic parameter for comparing protein hydrolysis processes. However, DH alone does not provide detailed information on the relative abundance of peptides within a defined molecular-weight region, nor does it describe how the peptide population is distributed across different size ranges [14]. For bioactive peptides, functional behavior is closely associated with molecular-weight distribution, amino acid composition, sequence structure, and conformational exposure [16]. Therefore, process evaluation based only on the overall hydrolysis extent may overlook changes in peptide-distribution profiles, especially when insufficient or excessive hydrolysis alters the accumulation of peptides within a predefined distribution window [10].

To address these limitations, the present study proposes a gel filtration chromatography (GFC)-guided sequential hydrolysis strategy based on the peak area of a defined distribution region [17]. Unlike optimization aimed simply at increasing DH, GFC reflects the molecular-weight distribution of hydrolysates through elution behavior. By integrating the peak area within a target region, the relative generation of major peptide components in a specified distribution window can be evaluated [18]. On this basis, papain and alcalase were applied in a stepwise hydrolysis process, and the integrated area of the GFC target window was used as the response variable for Box–Behnken response surface optimization [10,18]. It should be emphasized that this target window was not designed to represent a purified bioactive peptide fraction or to directly quantify specific antioxidant peptides. Instead, it was used as an operational process index to monitor the relative accumulation of soluble peptide components within a defined molecular-weight distribution range. The central rationale was to supplement, rather than replace, conventional DH-based evaluation with a distribution-oriented index, thereby shifting part of the process assessment from overall hydrolysis extent to peptide-distribution regulation. This approach may help identify conditions that favor peptide accumulation within the selected distribution window while avoiding distributional shifts associated with insufficient or excessive hydrolysis, and provide a more specific process-level basis for preparing *S. oualaniensis* protein hydrolysates [19,20].

Accordingly, *S. oualaniensis* protein was used as the substrate to establish a papain–alcalase sequential hydrolysis system guided by the GFC target-window peak area. Single-enzyme hydrolysates were prepared as controls, and DH was measured in the three comparative hydrolysates to distinguish overall peptide-bond cleavage from distribution within the GFC target window. Three questions were addressed: (1) whether $A_{\mathrm{target}}$ can serve as an operational index for characterizing peptide-distribution regulation while remaining complementary to DH; (2) whether the selected papain–alcalase sequence produces a peptide-composition profile distinct from those of the tested single-enzyme hydrolysates under the same total enzyme dosage and hydrolysis time; and (3) whether the resulting structural and compositional differences are accompanied by differences in solubility, relative in vitro radical-scavenging capacity, and emulsion stability. The functional assays were used only for post-optimization comparison and were not response variables in the RSM design.

To clarify the experimental workflow, the study proceeded in four stages. First, single-factor experiments were conducted to screen the pH of each enzymatic stage and to determine suitable ranges for the total enzyme dosage, papain/alcalase mass ratio, and total hydrolysis time of the sequential papain–alcalase process, using $A_{\mathrm{target}}$ as the evaluation index. Second, after fixing the pH conditions of the two enzymatic stages, the total enzyme dosage, papain/alcalase mass ratio, and total hydrolysis time were optimized using a Box–Behnken design. Third, the optimized sequential hydrolysate (SPH-opt) was prepared under the selected conditions. Finally, the papain hydrolysate (SPH-Pap) and alcalase hydrolysate (SPH-Alc) were prepared as single-enzyme controls using the same total enzyme dosage and total hydrolysis time as SPH-opt. Four samples—the untreated ASP substrate, SPH-Pap, SPH-Alc, and SPH-opt—were subsequently compared in terms of DH, amino acid composition, peptide profile, structural and physicochemical characteristics, and functional properties. Thus, SPH-Pap and SPH-Alc served as post-optimization controls and were not included in the response surface optimization.

## 2. Materials and Methods

### 2.1. Materials and Reagents

Chilled specimens of *Sthenoteuthis oualaniensis* were purchased from Zhoushan Zhongshui Fishery Co., Ltd. (Zhoushan, Zhejiang, China). After removal of the viscera, skin, and inedible tissues, the mantle muscle was collected as the experimental material. Papain (Cat. No. G8430; enzymatic activity, 1.0 × 10^5^ U/g) and alcalase (Cat. No. B8360; enzymatic activity, 2.0 × 10^5^ U/g) were obtained from Beijing Solarbio Science & Technology Co., Ltd. (Beijing, China). Acetonitrile and trifluoroacetic acid, both of chromatographic grade, were purchased from Merck (Merck KGaA, Darmstadt, Germany). Blue dextran 2000, used as the void-volume marker for GFC, and reagents for antioxidant assays, including DPPH and ABTS, were purchased from Sigma-Aldrich (St. Louis, MO, USA). The SDS-PAGE gel preparation kit was supplied by Epizyme Biotech Co., Ltd. (Shanghai, China; Cat. No. PG112). Unless otherwise stated, all other reagents were commercially available and of analytical grade.

### 2.2. Preparation of Sthenoteuthis oualaniensis Protein (ASP)

*Sthenoteuthis oualaniensis* protein (ASP) was prepared according to the method of Moghadam et al. [21], with minor modifications. The mantle muscle was minced and mixed with ice-cold 0.5% (*w*/*v*) NaHCO_3_ solution at a solid-to-liquid ratio of 1:3 (*w*/*v*). The mixture was stirred gently in an ice-water bath for 10 min to remove part of the lipids, water-soluble non-target proteins, and flavor-related low-molecular-weight compounds. The resulting homogenate was then centrifuged at 4000× *g* for 20 min, after which the supernatant was discarded and the precipitate was collected.

The precipitate was washed with pre-cooled deionized water at the same solid-to-liquid ratio, mixed thoroughly, and centrifuged again to reduce the influence of residual alkali and soluble impurities on subsequent enzymatic hydrolysis. The washed precipitate was spread evenly on stainless-steel trays, with the sample thickness controlled below 1 cm. Samples were pre-frozen at −80 °C for 12 h and then lyophilized using an HXLG-18-50B vacuum freeze dryer (Shanghai Huxi Industrial Co., Ltd., Shanghai, China). After the cold-trap temperature reached −40 °C, vacuum was applied, and drying was continued for 48 h at a chamber pressure below 10 Pa. The dried material was ground and passed through a 60-mesh sieve to obtain ASP powder, which was sealed and stored at −20 °C until further use.

Isoelectric precipitation was not applied because the present study aimed to evaluate the enzymatic hydrolysis of a mildly washed muscle-protein preparation rather than to prepare a purified protein isolate. Introducing a pH-shift and isoelectric-precipitation step would alter the initial substrate and introduce an additional pretreatment variable, which could confound comparison of the subsequent hydrolysis conditions. Therefore, a standardized washing and freeze-drying procedure was used to prepare the ASP substrate.

### 2.3. Preparation of Sthenoteuthis oualaniensis Protein Hydrolysates (SPH) and GFC-Guided Optimization

#### 2.3.1. Basic Procedure for Sequential Enzymatic Hydrolysis

*Sthenoteuthis oualaniensis* protein hydrolysates were prepared using papain–alcalase sequential hydrolysis. On the basis of preliminary experiments, the substrate concentration was fixed at 10% (*w*/*v*), the hydrolysis temperature was fixed at 55 °C, and papain hydrolysis was conducted before alcalase hydrolysis. The temperature of 55 °C was selected based on preliminary experiments and the compatible working ranges of papain and alcalase. This setting allowed the effects of total enzyme dosage, papain/alcalase ratio, and total hydrolysis time on the GFC target-window peak area to be evaluated under a controlled thermal condition. Therefore, temperature was not included as an independent variable in the Box–Behnken design of the present study and is considered a factor for future process refinement. The hydrolysis time ratio between the two stages was fixed at 3:1; that is, the papain stage accounted for 75% of the total hydrolysis time, whereas the alcalase stage accounted for 25%. The specific pH values, total enzyme dosage, papain/alcalase ratio, and total hydrolysis time were determined through subsequent single-factor experiments and response surface optimization.

Briefly, ASP powder was dispersed in deionized water to prepare a 10% (*w*/*v*) substrate suspension. For the pH-screening experiments, the system pH was adjusted to the designated initial value using 0.1 M HCl or NaOH immediately before papain addition. The pH was not actively maintained, and no additional acid or base was added during the papain hydrolysis stage. Before the second hydrolysis stage, the pH was readjusted to the designated value using 0.1 M HCl or NaOH, after which alcalase was added. No further pH correction was performed during the alcalase stage. Following the pH-screening experiments, the initial pH values for the papain and alcalase stages were fixed at 7.0 and 8.0, respectively. After hydrolysis and enzyme inactivation, the hydrolysates were centrifuged, and the supernatants were filtered through a 0.45 μm aqueous membrane and lyophilized under the same pre-freezing and freeze-drying conditions described in Section 2.2.

#### 2.3.2. Single-Factor Experiments

To screen process parameters affecting the generation of major peptide components within the target window, the GFC target-window peak area was used as the evaluation index. The effects of papain pH, alcalase pH, total enzyme dosage, papain/alcalase ratio, and total hydrolysis time on hydrolysate distribution were examined sequentially. Except for the factor under investigation, all other conditions were kept constant to ensure comparability among treatments. The pH values for papain were set at 6.0, 6.5, 7.0, 7.5, and 8.0, whereas the pH values for alcalase were set at 7.0, 7.5, 8.0, 8.5, and 9.0. The total enzyme dosage was set at 840, 980, 1120, 1260, and 1400 U/g dry ASP. The papain/alcalase ratio refers to the mass ratio of the two commercial enzyme preparations. For a dry ASP mass Ms (g), a target total enzyme dosage D (U/g), a papain/Alcalase mass ratio a:b, and declared activities UP = 1.0 × 105 U/g and UA = 2.0 × 105 U/g, the enzyme masses were calculated as mP = D × Ms × a/(UP × a + UA × b) and mA = D × Ms × b/(UP × a + UA × b). At the optimized setting (D = 1110 U/g; a:b = 3:5), 2.562 mg papain and 4.269 mg alcalase were added per gram of dry ASP. Accordingly, 25.62 mg papain and 42.69 mg alcalase were added per 10 g of dry ASP.

Because papain and alcalase differed in enzymatic activity, the papain/alcalase ratio was expressed as the percentage of papain mass in the total mass of the two added enzymes, with the corresponding mass ratio given in parentheses. The ratio gradients were set at 12.5% (1:7), 25.0% (1:3), 37.5% (3:5), 50.0% (1:1), and 62.5% (5:3). In this study, the total enzyme dosage denoted the total nominal enzymatic activity of the two proteases added per gram of dry ASP substrate, expressed as U/g. When total hydrolysis time was examined, the papain-to-alcalase hydrolysis time ratio was kept at 3:1. The total hydrolysis times were set at 4, 6, 8, 10, and 12 h, corresponding to papain/alcalase hydrolysis times of 3/1, 4.5/1.5, 6/2, 7.5/2.5, and 9/3 h, respectively.

#### 2.3.3. Evaluation of the GFC Target-Window Integrated Area

For GFC analysis, the freeze-dried hydrolysate powders obtained after centrifugation and 0.45 μm membrane filtration were redissolved in ultrapure water at a concentration of 2.0 mg/mL by dissolving 10 mg of each sample in 5.0 mL of ultrapure water. To remove residual particulates and prevent blockage of the gel-filtration column, the reconstituted sample solutions were passed again through a 0.45 μm aqueous microporous membrane filter. No additional centrifugation was performed before GFC analysis. A 2.0 mL aliquot of each filtrate was carefully loaded onto a Sephadex G-25 gel-filtration column (2.0 cm i.d. × 40 cm bed height). The column was connected to an MB99-1 automatic liquid chromatography separation system equipped with an HD-3 UV detector and an HD-A single-channel chromatogram acquisition unit (Shanghai Huxi Analysis Instrument Factory Co., Ltd., Shanghai, China). Ultrapure water was used as the mobile phase at a flow rate of 0.7 mL/min. Effluent absorbance at 220 nm was monitored continuously throughout elution and recorded using the HD-A acquisition unit. To reduce the influence of batch-to-batch variation in elution volume on peak-area comparison, the elution volume was normalized using the partition coefficient (K_av_), calculated as follows:

(1) $$K_{\mathrm{av}}=\frac{\left(V_{e}-V_{0}\right)}{\left(V_{t}-V_{0}\right)}$$

where Ve is the elution volume, V0 is the void volume determined using blue dextran 2000, and Vt is the total column-bed volume.

According to the elution distribution observed in preliminary experiments, K_av_ = 0.08–0.90 was defined as the operational target integration window. This window was used mainly to exclude interference from macromolecular proteins or aggregates eluting near the void volume (K_av_ ≈ 0), as well as free amino acids, salts, and extremely small molecular components eluting near the total column volume (K_av_ ≈ 1) [22,23]. The selected window was therefore used to represent the relative generation of major peptide components during hydrolysis. The area under the 220 nm absorbance curve within the target window was defined as the target integrated value ($A_{\mathrm{target}}$) and used as the response variable for response surface optimization. This window was used for process optimization and peptide-distribution comparison and should not be interpreted as a purification range for a single bioactive peptide fraction. Accordingly, $A_{\mathrm{target}}$ was used only to compare relative changes in the major soluble peptide region under different enzymatic hydrolysis conditions and was not regarded as direct evidence for the content of a specific antioxidant peptide or a single active component.

(2) $$A_{\mathrm{target}}=\int_{0.08}^{0.9}{\;\mathrm{Abs}}_{220}nm\left(K_{\mathrm{av}}\right){\mathrm{dK}}_{\mathrm{av}}$$

#### 2.3.4. Response Surface Methodology (RSM) Optimization

On the basis of the single-factor experiments, three factors that markedly affected the GFC target-window peak area were selected for response surface optimization: total enzyme dosage (A), papain/alcalase ratio (B), and total hydrolysis time (C). A three-factor, three-level Box–Behnken design (BBD) was applied, with the GFC target-window integrated area (Y) used as the response variable. A quadratic polynomial regression model was established to determine the preferred process conditions. The factors and levels are listed in Table 1.

#### 2.3.5. Preparation of the Optimized Hydrolysate (SPH-opt) and Single-Enzyme Controls (SPH-Pap and SPH-Alc)

The sequential two-enzyme hydrolysate was prepared according to the response surface optimization results and was designated SPH-opt. To compare the effects of different enzymatic hydrolysis modes on hydrolysate structure and functional properties, a single papain hydrolysate (SPH-Pap) and a single alcalase hydrolysate (SPH-Alc) were prepared in parallel as controls. The total enzyme dosage and total hydrolysis time for the single-enzyme controls were kept the same as those used for SPH-opt. SPH-Pap was hydrolyzed only at the pH set for papain, whereas SPH-Alc was hydrolyzed only at the pH set for alcalase. After hydrolysis, enzyme inactivation, centrifugation, filtration, and freeze-drying were performed as described in Section 2.3.1. Three hydrolysates, namely SPH-opt, SPH-Pap, and SPH-Alc, were finally obtained for subsequent structural characterization and functional-property evaluation.

#### 2.3.6. Determination of the Degree of Hydrolysis

The degree of hydrolysis (DH) of SPH-Pap, SPH-Alc, and SPH-opt was estimated using the improved o-phthaldialdehyde (OPA) method of Nielsen et al. [24], with calculation parameters previously applied to squid protein hydrolysates [25]. The OPA working reagent was prepared by dissolving 3.810 g sodium tetraborate decahydrate and 0.100 g SDS in deionized water, adding 0.080 g OPA dissolved in 2.00 mL ethanol and 0.088 g dithiothreitol, and making the final volume to 100 mL. The L-serine standard was 0.9516 meq/L. Each freeze-dried hydrolysate was dissolved at 1.0 mg/mL. SPH-Alc and SPH-opt were diluted twofold before the OPA reaction to maintain absorbance close to the standard-response range, and the dilution factor was included in the calculation. Thus, the dilution factor D was 1 for SPH-Pap and 2 for SPH-Alc and SPH-opt. For each determination, 3.00 mL of OPA reagent was mixed with 400 μL of sample solution, L-serine standard, or deionized water (blank), vortexed for 5 s, allowed to react at room temperature for exactly 2 min, and measured at 340 nm using a P4 UV–visible spectrophotometer (Shanghai Mapada Instruments Co., Ltd., Shanghai, China).

Because the undiluted hydrolysate solutions were prepared at 1.0 mg/mL, the original Nielsen equation was simplified as follows:

(3) $${\mathrm{Serine}-\mathrm{NH}}_{2}=\frac{A_{\mathrm{sample}}\;-\;A_{\mathrm{blank}}}{A_{\mathrm{standard}}\;-\;A_{\mathrm{blank}}}\times \frac{95.16D}{P_{\mathrm{eq}}}$$

(4) $$h=\frac{{\mathrm{Serine}-\mathrm{NH}}_{2}-\beta }{\alpha }$$

(5) $$\mathrm{DH}=\frac{h}{h_{\mathrm{tot}}}\times \;100\%$$

(6) $$P_{\mathrm{eq}}=\Sigma_{i}C_{i}\frac{M_{i}\;-\;18.015}{M_{i}}$$

where Serine-NH_2_ is the serine amino equivalent (meq/g protein equivalent); *A*_sample_, *A*_blank_, and *A*_standard_ are the absorbances of the sample, reagent blank, and L-serine standard, respectively; *D* is the dilution factor; *P*_eq_ is the protein-equivalent content expressed as a percentage; *h* is the number of hydrolyzed peptide bonds per protein equivalent; *h*_tot_ is the total number of peptide bonds per protein equivalent; and α and β are protein-dependent calibration constants. Values of α = 1.00, β = 0.40, and *h*_tot_ = 8.6 meq/g protein equivalent were used, following the fish-protein constants reported by Nielsen et al. and their subsequent application to squid protein hydrolysates [24,25].

Because total-nitrogen protein data were unavailable, *P*_eq_ was estimated consistently for the three hydrolysates from the experimentally determined amino acid composition on an amino-acid-residue basis, where *C*_i_ is the measured content of amino acid *i* (g/100 g sample) and *M*_i_ is its molecular mass. The resulting *P*_eq_ values were 70.20%, 65.85%, and 68.97% for SPH-Pap, SPH-Alc, and SPH-opt, respectively. Serine standards and reagent blanks were measured before and after each sample series, and their mean absorbance values were used. For each hydrolysate, three independent processing batches were prepared and analyzed separately by the OPA method; the corresponding dilution factor was included where applicable.

### 2.4. Structural and Compositional Characterization

#### 2.4.1. Amino Acid Composition

The amino acid compositions of ASP, SPH-Pap, SPH-Alc, and SPH-opt were determined using an LA8080 high-speed amino acid analyzer (Hitachi High-Tech Corporation, Tokyo, Japan), with reference to Akbarbaglu et al. [26] and with appropriate modifications. Approximately 30 mg of each sample was mixed with 10 mL of 6 M HCl containing 0.1% phenol, flushed with nitrogen for 1–2 min, sealed, and hydrolyzed at 85 °C for 72 h. After removal of HCl under reduced pressure, the residue was reconstituted to 10 mL and filtered through a 0.22 μm membrane. A 20 μL aliquot was separated on a 4.6 mm × 60 mm cation-exchange column using sodium citrate buffers at a flow rate of 0.40 mL/min and a column temperature of 57 °C. Post-column ninhydrin derivatization was performed at 135 °C with a reagent flow rate of 0.35 mL/min. Amino acids were detected at 570 nm, whereas proline was detected at 440 nm. Peaks were identified and quantified using a mixed amino acid standard, and the results were expressed as g/100 g sample. Total amino acids (TAA), EAA/TAA, EAA/NEAA, and HAA/TAA were subsequently calculated to compare the amino acid compositions of the samples.

#### 2.4.2. SDS-PAGE

Tricine-SDS-PAGE was used to analyze the degree of protein degradation and molecular-weight distribution of ASP and its hydrolysates. To observe both high-molecular-weight protein bands and low-molecular-weight peptide distributions, 12% and 16.5% separating gels were used [27]. The 12% separating gel, together with a 10–250 kDa protein marker, was used to observe the degradation of major high-molecular-weight protein bands in ASP and the hydrolysates. The 16.5% high-concentration separating gel, together with a 2.7–40 kDa low-molecular-weight marker, was used to further analyze the distribution of low-molecular-weight peptides.

ASP, SPH-Pap, SPH-Alc, and SPH-opt were each prepared as sample solutions at the same mass concentration. Each sample solution was mixed with 5× loading buffer at a ratio of 4:1 (*v*/*v*), heated at 100 °C for 10 min, and centrifuged. The supernatant was then loaded onto the gel. The loading volume was 10 μL per sample lane and 5 μL for the marker. Electrophoresis was first conducted at 80 V through the stacking gel. After bromophenol blue entered the separating gel, the voltage was increased to 120 V until the indicator band migrated close to the bottom of the gel. After electrophoresis, the 12% gel was stained with Coomassie Brilliant Blue, whereas the 16.5% gel was developed using a high-sensitivity staining method. After staining and destaining, the gels were imaged using an Amersham Imager 600 system (Cytiva, Marlborough, MA, USA).

#### 2.4.3. Fourier Transform Infrared Spectroscopy (FTIR)

FTIR analysis was performed according to the method of Luo et al. [28], with slight modifications. Freeze-dried samples (ASP, SPH-Pap, SPH-Alc, and SPH-opt) were mixed with dry KBr powder at a ratio of 1:100 (*w*/*w*), ground thoroughly, and pressed into tablets. Spectra were collected using a Fourier transform infrared spectrometer (Nicolet 6700, Thermo Fisher Scientific, Waltham, MA, USA) over the range of 4000–400 cm^−1^ at a resolution of 4 cm^−1^. Each sample was scanned 32 times, and a blank KBr tablet was used as the background. Changes in the amide A, amide I, and amide II bands were analyzed. The amide I band in the range of 1600–1700 cm^−1^ was baseline-corrected and subjected to Gaussian fitting to estimate the relative contents of secondary structures in different samples.

#### 2.4.4. Ultraviolet Absorption Spectroscopy (UV)

UV spectroscopy was performed according to the method of Wang et al. [29], with appropriate modifications. Freeze-dried powders of ASP, SPH-Pap, SPH-Alc, and SPH-opt were separately weighed and dissolved in deionized water to prepare 0.5 mg/mL sample solutions. Because ASP showed poor dispersibility in water, the ASP solution was centrifuged before measurement, and the supernatant was used for analysis. Other hydrolysate solutions were treated in the same manner when turbidity was observed. Deionized water was used as the blank, and spectra were recorded from 200 to 400 nm at 1 nm intervals using a P4 UV–visible spectrophotometer (Shanghai Mapada Instruments Co., Ltd., Shanghai, China). Changes in absorbance near 231 and 280 nm were recorded to evaluate the effects of enzymatic hydrolysis on peptide-backbone absorption and exposure of aromatic amino acid residues.

#### 2.4.5. Differential Scanning Calorimetry (DSC)

The thermal-transition behavior of ASP, SPH-Pap, SPH-Alc, and SPH-opt was determined using a differential scanning calorimeter (DSC 200 F3, NETZSCH-Gerätebau GmbH, Selb, Germany), following the method of Khan et al. [30] with minor modifications. An appropriate amount of freeze-dried sample was placed in an aluminum crucible, compacted, and sealed, with an empty crucible used as the reference. Scanning was performed under high-purity nitrogen at a flow rate of 20 mL/min. The temperature range was 30–160 °C, and the heating rate was 10 °C/min. Heat-flow curves were recorded, and the peak temperature (Tp) and enthalpy change (ΔH) were analyzed to compare the effects of different enzymatic hydrolysis treatments on protein thermal stability.

#### 2.4.6. LC-MS/MS-Based Peptidomic Analysis

Peptide compositions of different hydrolysates were analyzed by LC-MS/MS, and peptide sequence analysis was conducted according to the method of Sun et al. [31], with slight modifications. Freeze-dried SPH-Pap, SPH-Alc, and SPH-opt samples were redissolved and passed through 10 kDa ultrafiltration tubes to remove residual macromolecular components. The filtrates were desalted using C18 ZipTips and dried before analysis. After reconstitution, samples were analyzed using an Easy-nLC 1200 nano-liquid chromatography system coupled with a Q-Exactive Plus mass spectrometer (Thermo Fisher Scientific, Waltham, MA, USA). Peptides were separated on a C18 nano-column. Mobile phase B consisted of acetonitrile containing 0.1% formic acid, and the elution gradient increased from 5% to 35% B over 43 min at a flow rate of 300 nL/min.

Mass spectrometric acquisition was performed in data-dependent acquisition (DDA) mode. Precursor ions with relatively high abundance were selected for HCD fragmentation, with a normalized collision energy (NCE) of 27. Raw data were searched against the *Sthenoteuthis oualaniensis* protein database using Proteome Discoverer. The digestion mode was set as unspecific, and the mass tolerances for precursor and fragment ions were set at 10 ppm and 0.02 Da, respectively. The obtained peptide sequences were used to compare the effects of different hydrolysis modes on short-peptide release and the composition of peptides containing aromatic residues. Peptide identifications were filtered according to the high-confidence results exported by Proteome Discoverer, and relative peptide signals were used mainly to compare compositional differences among hydrolysates. Relative MS response ratios between SPH-opt and SPH-Alc were calculated directly from the peptide-intensity values exported by Proteome Discoverer; peptides not detected under the present LC-MS/MS conditions were reported as ND. These LC-MS/MS data were used for exploratory comparative profiling; no inferential statistical analysis or absolute peptide quantification was performed using peptide-intensity values.

### 2.5. Characterization of Physical Properties

#### 2.5.1. Particle-Size Distribution and Zeta Potential

Particle-size distribution and zeta potential were determined according to the method of Zhu et al. [32], with appropriate modifications. Because ASP and the hydrolysates differed markedly in particle scale, different instruments were used. The particle-size distribution of ASP was measured using a laser particle-size analyzer (Mastersizer 3000, Malvern Instruments Ltd., Malvern, Worcestershire, UK). The particle sizes and zeta potentials of SPH-Pap, SPH-Alc, and SPH-opt were measured using a nano-particle-size and zeta-potential analyzer (Zetasizer Nano ZS90, Malvern Instruments Ltd., Malvern, Worcestershire, UK).

Before measurement, all samples were fully dispersed in deionized water at the same mass concentration and diluted appropriately according to instrument requirements. All measurements were performed at 25 °C. Particle-size distribution was used to evaluate the effects of enzymatic hydrolysis on protein particle scale and dispersion state, whereas zeta potential was used to characterize the surface charge properties of the hydrolysates.

#### 2.5.2. Rheological Properties

Apparent viscosity was measured because flow behavior is closely related to the processing applicability of protein hydrolysates in liquid and semi-liquid food systems. In the present study, viscosity was not used as a direct bioactivity indicator, but as a processing-related physical parameter to evaluate whether enzymatic hydrolysis improved the dispersibility and flowability of *S. oualaniensis* protein dispersions. The apparent viscosities of ASP, SPH-Pap, SPH-Alc, and SPH-opt were measured using a rotational rheometer (MCR 302, Anton Paar GmbH, Graz, Austria), according to the method of Chen et al. [33] with appropriate modifications. Samples were prepared as dispersions at the same mass concentration, thoroughly mixed, and degassed before measurement. A 25 mm parallel-plate geometry was used. After equilibration at 25 °C for 2 min, steady shear scanning was performed over a shear-rate range of 0.1–1000 s^−1^. Changes in apparent viscosity as a function of shear rate were recorded to compare the effects of enzymatic hydrolysis treatments on sample flow behavior.

### 2.6. Evaluation of Functional Properties

#### 2.6.1. Solubility

Solubility was determined according to the method of Huang et al. [34], with appropriate modifications. ASP, SPH-Pap, SPH-Alc, and SPH-opt were separately prepared as 1% solutions. The pH values were adjusted to 2, 4, 6, 8, 10, and 12 using 0.1 M HCl or NaOH. After stirring at room temperature for 30 min, samples were centrifuged, and the supernatants were collected. Protein contents before centrifugation and in the supernatants after centrifugation were measured using a BCA assay kit (BL521A, Biosharp, Beijing, China) to calculate solubility.

#### 2.6.2. Relative In Vitro Radical-Scavenging Capacity

DPPH and ABTS radical-scavenging capacities were measured according to the method of Dong et al. [35], with appropriate modifications. ASP, SPH-Pap, SPH-Alc, and SPH-opt were prepared at 0.3125, 0.625, 1.25, 2.5, and 5.0 mg/mL and reacted with DPPH ethanol solution or ABTS working solution. Sample background controls and blank controls were included to correct for sample color and solvent background. No external antioxidant standard such as Trolox, BHT, or ascorbic acid was included. Accordingly, the results were used only to compare relative radical-scavenging capacity among the prepared samples under the present assay conditions and not to benchmark absolute antioxidant potency. The DPPH system was incubated in the dark for 30 min and measured at 517 nm, whereas the ABTS system was reacted for 6 min and measured at 734 nm using an HBS-ScanX full-wavelength microplate reader (Nanjing Detie Biotechnology Co., Ltd., Nanjing, China). Radical-scavenging rates were calculated from the corrected sample, sample-background, and blank-control absorbance values.

#### 2.6.3. Emulsifying Properties and Emulsion Stability

The emulsifying activity index (EAI) and emulsion stability index (ESI) were determined according to the method of Yang et al. [36], with appropriate modifications. Sample solution and soybean oil were mixed at a ratio of 3:1 (*v*/*v*) and homogenized at 10,000 rpm for 1 min using a T 10 basic ULTRA-TURRAX disperser equipped with an S25 dispersing element (IKA-Werke GmbH & Co. KG, Staufen, Germany). Aliquots were collected from the bottom of the emulsion immediately after homogenization and after standing for 10 min. The absorbance values measured at 0 and 10 min were denoted A0 and A10, respectively. The collected samples were diluted with 0.1% (*w*/*v*) sodium dodecyl sulfate (SDS) solution. The SDS solution was used as the blank for zero adjustment, and absorbance was measured at 500 nm. EAI and ESI were then calculated.

### 2.7. Statistical Analysis

Unless otherwise stated, each hydrolysis treatment was prepared in three independent processing batches. For each independently prepared batch, analytical measurements were performed under the same conditions. Results are expressed as mean ± standard deviation (mean ± SD) of three independent processing replicates. One-way analysis of variance (ANOVA) and Tukey’s multiple-comparison test were performed using SPSS Statistics 22.0 (IBM Corp., Armonk, NY, USA), and *p* < 0.05 was considered statistically significant. Response surface experimental design and data analysis were performed using Design-Expert 13.0 (Stat-Ease Inc., Minneapolis, MN, USA). K_av_ normalization of raw GFC data and target-window integration were conducted using MATLAB R2025a (The MathWorks Inc., Natick, MA, USA), and figures were prepared using Origin 2025 (OriginLab Corporation, Northampton, MA, USA).

## 3. Results and Discussion

### 3.1. Amino Acid Profiles of ASP and Its Hydrolysates

As shown in Table 2, ASP and the three hydrolysates all retained a relatively complete amino acid profile, with total amino acid contents ranging from 76.89 to 82.00 g/100 g. Glutamic acid, aspartic acid, leucine, lysine, alanine, and arginine were the predominant amino acids across all samples, indicating that the major amino acid pattern was broadly maintained after hydrolysis. The essential amino acid ratio (EAA/TAA) ranged from 38.18% to 39.20% and varied only slightly among the samples. Compared with ASP, only minor changes in EAA/TAA were observed for SPH-Pap, SPH-Alc, and SPH-opt, indicating that the enzymatic hydrolysis modes used in this study did not substantially alter the overall proportion of essential amino acids in *S. oualaniensis* protein. At the individual-amino-acid level, lysine was highest in SPH-Pap (8.33 g/100 g) and lower in SPH-Alc and SPH-opt (6.84 and 7.21 g/100 g, respectively) than in ASP (7.42 g/100 g), whereas methionine ranged from 0.35 g/100 g in SPH-Pap to 0.65 g/100 g in SPH-Alc, with SPH-opt (0.50 g/100 g) remaining close to ASP (0.53 g/100 g). These differences indicate modest redistribution of individual essential amino acids among the prepared hydrolysates rather than a uniform increase or decrease. It should be noted, however, that valine and the sulfur-containing amino acids (Met + Cys) were below the FAO/WHO reference values in all samples, whereas isoleucine was slightly below the reference value in SPH-Pap and SPH-Alc [37]. The present amino acid data are therefore used for descriptive within-study comparison and preliminary nutritional assessment rather than for making broader claims about overall protein nutritional quality.

The hydrophobic amino acid ratio (HAA/TAA) remained within a narrow range of 36.05–37.63%, with SPH-opt showing a value of 37.47%. Given the generally small differences in amino acid composition among the treatments, subsequent changes in functional properties should not be attributed simply to changes in total amino acid composition. Such changes are more likely associated with hydrolysis-induced differences in peptide length, sequence composition, and residue exposure [38]. Of particular relevance, hydrophobic or aromatic amino acids such as leucine, alanine, tyrosine, and phenylalanine were still present at appreciable levels in SPH-opt. Aromatic residues may be relevant to radical-scavenging behavior through hydrogen donation, electron transfer, or radical-stabilizing effects [39].

### 3.2. Preliminary Screening of Enzymatic Hydrolysis Conditions

**Figure 1:**
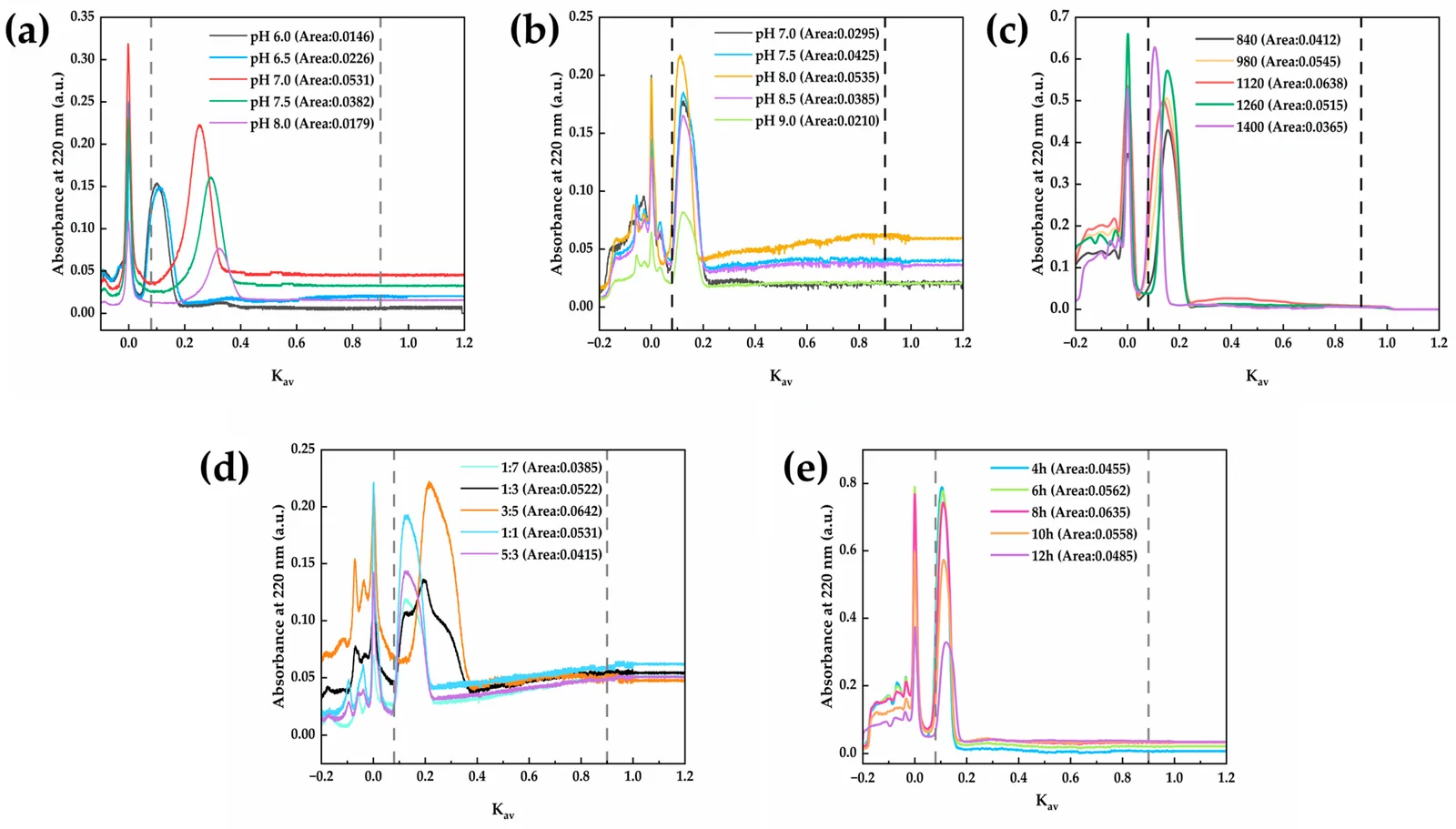
GFC profiles of *Sthenoteuthis oualaniensis* protein hydrolysates prepared under different single-factor enzymatic hydrolysis conditions. (**a**) Papain pH; (**b**) alcalase pH; (**c**) total enzyme dosage; (**d**) papain/alcalase mass ratio; (**e**) total hydrolysis time. The dashed lines indicate the target integration window of Kₐᵥ = 0.08–0.90. Representative GFC profiles are shown; target-window peak areas were calculated from three independent processing batches.

pH affects both the charge state of protease active centers and the dissociation behavior of substrate proteins; thus, pH is a fundamental parameter that must be determined first in a sequential hydrolysis system [14,40]. As shown in Figure 1a,b, the papain-treated system produced a relatively high GFC target-window peak area at pH 7.0, whereas the alcalase-treated system performed better at pH 8.0. Once the pH deviated from the suitable range, the target-window peak area decreased, indicating that the relative accumulation of 220 nm absorbing components within the target window decreased. Therefore, the reaction pH values for papain and alcalase were fixed at 7.0 and 8.0, respectively, for subsequent experiments. Although papain and alcalase are both broad-specificity proteases, their catalytic performance and substrate accessibility are still affected by pH. Therefore, different pH values were used for the two stages to allow each enzyme to act under conditions that favored the generation of peptides within the GFC target window. The use of different pH values was intended to optimize the stepwise hydrolysis process rather than to prove a direct synergistic interaction between the two enzymes.

Both total enzyme dosage and hydrolysis time showed an initial increase followed by a decrease in the GFC target-window peak area (Figure 1c,e). When the total enzyme dosage increased to 1120 U/g dry ASP, the target-window peak area reached 0.0630. When the total hydrolysis time was extended to 8 h, the target-window peak area reached 0.0638. These results indicate that an appropriate increase in enzyme dosage and hydrolysis time favored the accumulation of soluble 220 nm absorbing components within the selected target window [41]. However, further increases in enzyme dosage or prolonged hydrolysis reduced the target-window peak area, suggesting that some components generated earlier may have been further cleaved and shifted outside the defined GFC window. This behavior points to a balance between accumulation within the selected window and further cleavage during *S. oualaniensis* protein hydrolysis [42].

The effect of the papain/alcalase ratio on the target-window peak area is shown in Figure 1d. When the mass ratio of papain to alcalase was 3:5, the target-window peak area reached 0.0642, higher than those obtained with the other ratios. This finding indicates that an appropriate balance between the two enzymes favors the formation of peptide components within the target window [43]. This phenomenon may be associated with differences in cleavage preference and substrate accessibility between papain and alcalase [44]. Papain pretreatment may partially loosen the protein structure and expose additional cleavage sites, after which alcalase may further cleave accessible protein backbones and promote the release of peptides in the target region. However, because reverse-order and simultaneous dual-enzyme hydrolysis were not included in the present experimental design, this result should be interpreted as a complementary tendency under the selected papain–alcalase sequence rather than as direct evidence of enzymatic synergy or absolute order superiority.

### 3.3. Optimization by Response Surface Methodology

On the basis of the single-factor experiments, multiple regression fitting of the Box–Behnken design (BBD) data was performed using Design-Expert 13.0. Total enzyme dosage (A), papain/alcalase ratio (B), and hydrolysis time (C) were used as independent variables, and the GFC target-window peak area (Y) was used as the response variable. The Box–Behnken experimental design and observed responses are presented in Table 3. The quadratic polynomial regression equation based on the coded BBD levels was established as follows [45]:

(7) $$Y=0.0645-0.0019\;A+0.0003\;B-0.0004\;C+0.0015\;\mathrm{AB}-0.0019\;\mathrm{AC}+0.0012\;\mathrm{BC}-0.0104\;A^{2}-0.0114{\;B}^{2}-0.0078\;C^{2}$$

**Table 3:** Box–Behnken experimental design and observed GFC target-window peak areas.

Run	A: Total Enzyme Dosage (U/g Dry ASP)	B: Papain/Alcalase Mass Ratio	C: Hydrolysis Time (h)	Y: GFC Target-Window Peak Area
1	−1	−1	0	0.0454
2	1	−1	0	0.0388
3	−1	1	0	0.0438
4	1	1	0	0.0431
5	−1	0	−1	0.0471
6	1	0	−1	0.0470
7	−1	0	1	0.0493
8	1	0	1	0.0418
9	0	−1	−1	0.0467
10	0	1	−1	0.0441
11	0	−1	1	0.0442
12	0	1	1	0.0463
13	0	0	0	0.0672
14	0	0	0	0.0628
15	0	0	0	0.0652
16	0	0	0	0.0613
17	0	0	0	0.0662

Note: A represents total enzyme dosage; B represents the papain/alcalase mass ratio; C represents total hydrolysis time. Y represents the GFC target-window peak area. The observed Y values were calculated from independently prepared processing batches under each Box–Behnken design condition.

The ANOVA results of the fitted quadratic model are provided in Supplementary Table S1. The regression model was highly significant (model F = 43.67, *p* < 0.0001), whereas the lack-of-fit term was not significant (*p* = 0.9415). These results indicate that the model fitted the experimental data well. The coefficient of determination (R^2^), predicted R^2^, and adjusted R^2^ were 0.9825, 0.9512, and 0.9600, respectively, with only a small difference between the latter two values. The adequate precision value was 17.1704, confirming that the model had an acceptable signal-to-noise ratio and good predictive capacity.

**Figure 2:**
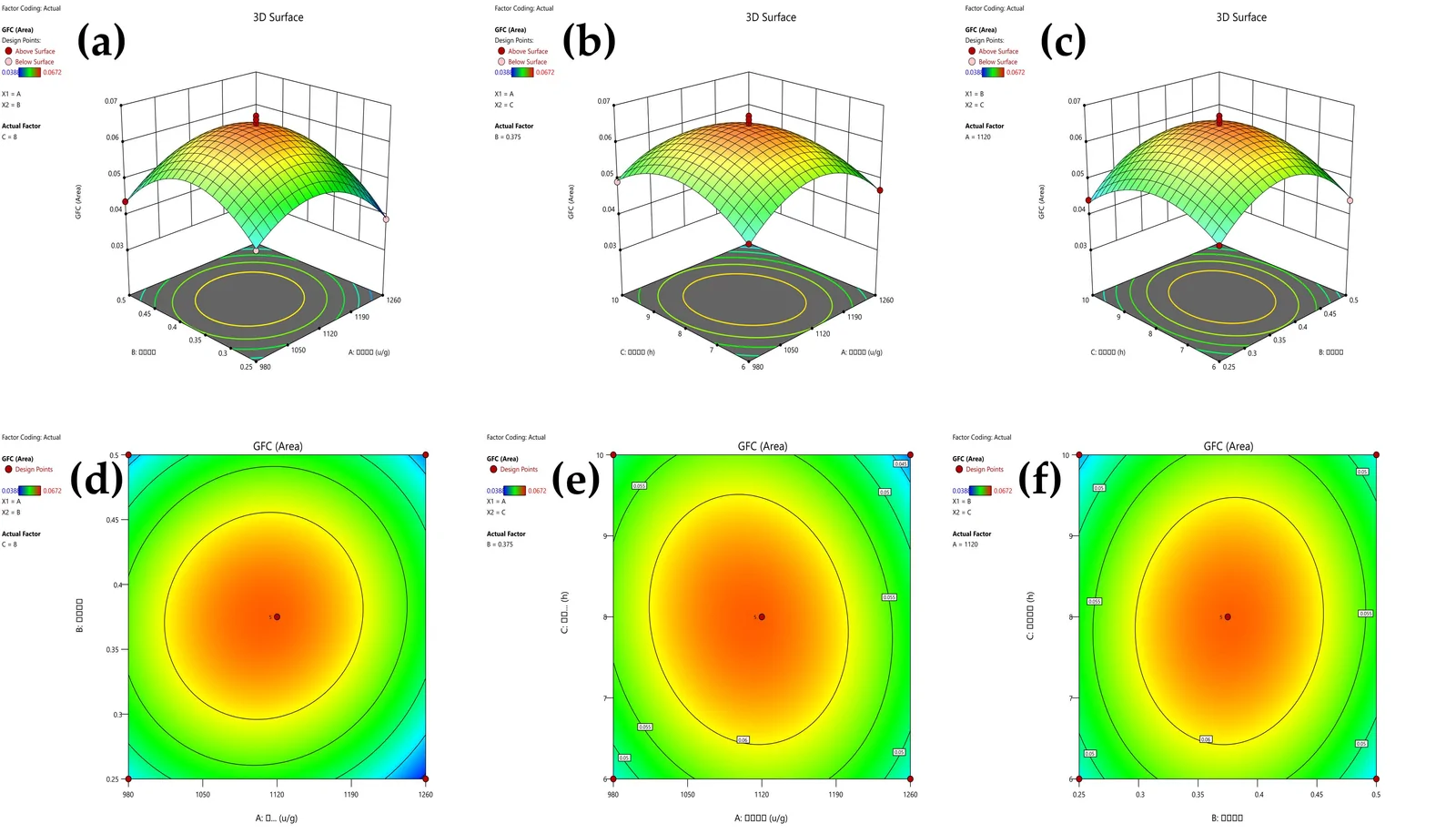
Response surface and contour plots showing the effects of enzymatic hydrolysis parameters on GFC target-window peak area. (**a**–**c**) Three-dimensional response surface plots for the interactions of total enzyme dosage, papain/alcalase mass ratio, and total hydrolysis time; (**d**–**f**) corresponding contour plots.

Regarding individual effects, the linear term of total enzyme dosage (A) had a significant influence on the response value (*p* = 0.0292), whereas the individual linear effects of papain/alcalase ratio (B) and hydrolysis time (C) were not significant. The interaction terms AB, AC, and BC also did not reach statistical significance. By contrast, the quadratic terms A^2^, B^2^, and C^2^ were all highly significant (*p* < 0.0001), indicating a pronounced nonlinear relationship between the GFC target-window peak area and the three factors. This pattern is consistent with the peak observed near the central region of the response surface in Figure 2: insufficient parameter levels did not promote adequate release of major peptide components, whereas excessive parameter levels likely caused further peptide degradation and reduced the target-window peak area.

Global optimization based on the fitted model gave a desirability value of 0.909. The predicted theoretical optimum was a total enzyme dosage of 1107.67 U/g dry ASP, a papain/alcalase ratio of 37.6%—close to 37.5%, corresponding to a 3:5 mass ratio—and a hydrolysis time of 7.968 h. Under these conditions, the predicted GFC target-window peak area was 0.065. For practical operability, the optimized conditions were adjusted to 1110 U/g dry ASP, a papain/alcalase mass ratio of 3:5, and 8 h. Verification experiments performed using three independent processing batches gave an $A_{\mathrm{target}}$ of 0.0648 ± 0.0012 a.u., in good agreement with the predicted value. $A_{\mathrm{target}}$ reflects the relative abundance of 220 nm absorbing soluble components within K_av_ = 0.08–0.90 and is therefore a peptide-distribution-oriented operational index [46,47]. It does not quantify overall peptide-bond cleavage, prove enrichment of a specific antioxidant peptide, or optimize radical-scavenging performance. The subsequent DH and functional measurements were used as independent comparative outcomes to clarify what information $A_{\mathrm{target}}$ adds beyond conventional hydrolysis extent.

### 3.4. Degree of Hydrolysis

The OPA-derived DH values of SPH-Pap, SPH-Alc, and SPH-opt were 10.72 ± 0.08%, 35.32 ± 0.12%, and 25.03 ± 0.37%, respectively (Table 4). The corresponding GFC target-window peak areas were 0.0528 ± 0.0015, 0.0485 ± 0.0018, and 0.0648 ± 0.0012 a.u., respectively. For both DH and $A_{\mathrm{target}}$, all pairwise differences among the three hydrolysates were significant (*p* < 0.05). SPH-Alc therefore showed the greatest apparent overall peptide-bond cleavage within the present comparison, whereas SPH-opt had an intermediate DH. Importantly, the highest DH did not coincide with the highest GFC target-window peak area: SPH-opt had the highest $A_{\mathrm{target}}$ despite a substantially lower DH than SPH-Alc. Within the present study, this mismatch supports treating DH and $A_{\mathrm{target}}$ as complementary rather than interchangeable indices. Previous studies of sequential hydrolysis have likewise shown that DH and peptide molecular-size distribution do not necessarily change in parallel [48]. Therefore, DH should not be regarded as a complete surrogate for peptide-size distribution.

The higher DH of SPH-Alc is qualitatively consistent with the larger number and total relative signal of short peptides observed by LC-MS/MS. More extensive hydrolysis can shift part of the peptide population toward very small peptides or free amino acids outside the defined GFC target window [10,42]. By contrast, the papain–alcalase sequence produced an intermediate overall DH while favoring the accumulation of soluble components within the selected distribution window. SPH-Alc had the highest DH, whereas SPH-opt showed the highest $A_{\mathrm{target}}$, broader pH solubility, moderately higher relative DPPH/ABTS scavenging rates, and higher emulsion stability. Taken together, these observations indicate that DH alone did not account for all between-sample differences in peptide distribution and functional outcomes. Nevertheless, because only three hydrolysis modes were compared, the associations among DH, $A_{\mathrm{target}}$, peptide composition, and functional properties should not be interpreted as statistical correlations or causal relationships.

### 3.5. Comparative Structural Characterization

#### 3.5.1. Molecular-Weight Distribution (SDS-PAGE)

**Figure 3:**
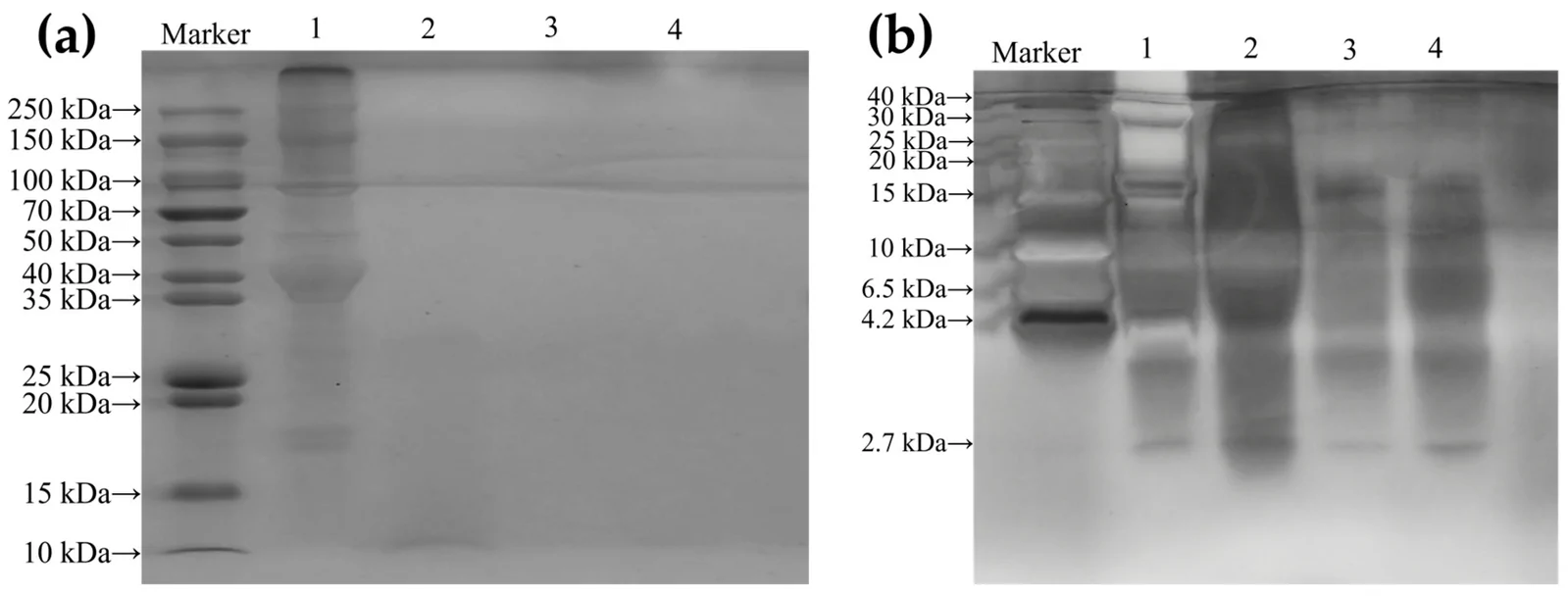
SDS-PAGE profiles of ASP and its hydrolysates prepared using different enzymatic hydrolysis modes. (**a**) Protein profiles obtained using 12% separating gel; (**b**) low-molecular-weight peptide profiles obtained using 16.5% separating gel. Lane 1, ASP; lane 2, SPH-Pap; lane 3, SPH-Alc; lane 4, SPH-opt. ASP, untreated *Sthenoteuthis oualaniensis* protein; SPH-Pap, papain hydrolysate; SPH-Alc, alcalase hydrolysate; SPH-opt, optimized sequential hydrolysate. Representative profiles from three independent processing batches are shown.

Sodium dodecyl sulfate–polyacrylamide gel electrophoresis (SDS-PAGE) was used to compare the effects of different enzymatic hydrolysis modes on the molecular-weight distribution of *S. oualaniensis* protein [49]. As shown in Figure 3a, the raw protein sample, ASP, displayed several distinct protein bands within the range of 35–250 kDa, indicating that ASP was mainly composed of high-molecular-weight myofibrillar proteins and related structural proteins [50]. After enzymatic hydrolysis, the high-molecular-weight bands of SPH-Pap, SPH-Alc, and SPH-opt were markedly weakened, suggesting that the protein backbone had undergone degradation to different extents. In particular, high-molecular-weight bands were almost absent in SPH-Alc and SPH-opt, indicating that the involvement of alcalase resulted in more extensive disruption of the macromolecular structure of *S. oualaniensis* protein.

In the low-molecular-weight region, SPH-Pap showed a broad diffuse distribution, suggesting that a certain amount of incompletely degraded intermediate fragments remained after single papain hydrolysis. By contrast, SPH-Alc and SPH-opt were mainly concentrated in the lower-molecular-weight region, indicating that alcalase facilitated further cleavage of intermediate fragments and promoted the formation of short peptides. Notably, SPH-opt showed a relatively continuous diffuse distribution in the low-molecular-weight region, together with limited residual medium- and high-molecular-weight components. The papain–alcalase sequential process therefore promoted macromolecular protein degradation and generated hydrolysates dominated by low-molecular-weight peptides. This result agrees with the GFC target-window peak-area optimization described above.

#### 3.5.2. FTIR Spectral Characteristics and Secondary-Structure Distribution

**Figure 4:**
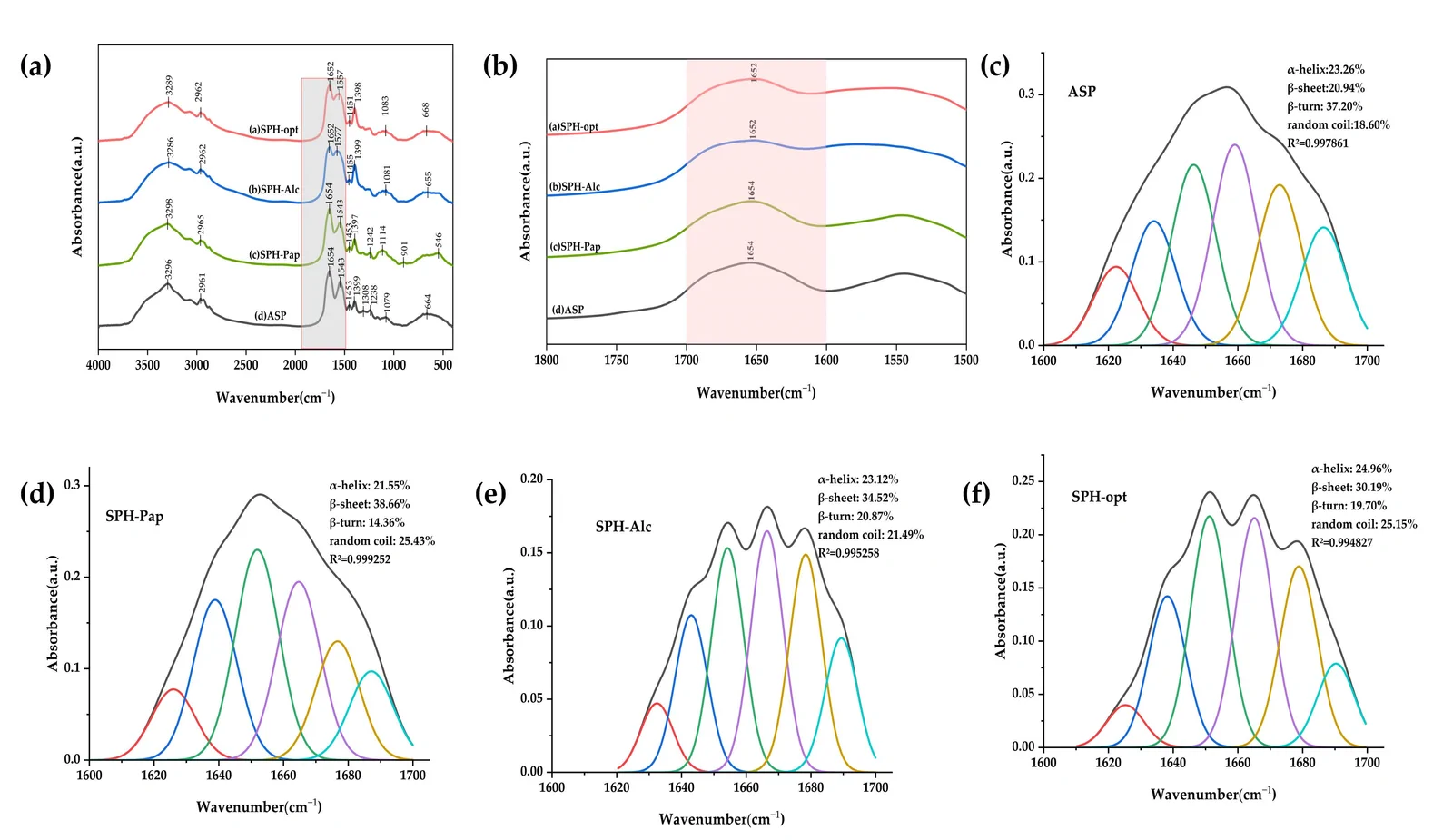
FTIR spectra and amide I band deconvolution of ASP and its hydrolysates. (**a**) Full FTIR spectra from 4000 to 500 cm^−1^; (**b**) enlarged spectra of the amide I region; (**c**–**f**) Gaussian deconvolution of the amide I band for ASP, SPH-Pap, SPH-Alc, and SPH-opt, respectively. In panels (**a**,**b**), the black, green, blue, and red curves represent ASP, SPH-Pap, SPH-Alc, and SPH-opt, respectively. In panels (**c**–**f**), the black curves represent the overall amide I spectral envelopes, whereas the colored curves represent the individual Gaussian-fitted component bands. Representative profiles from three independent processing batches are shown.

Fourier transform infrared spectroscopy (FTIR) was used to assess the effects of enzymatic hydrolysis on protein secondary structure and the hydrogen-bonding environment. As shown in Figure 4a,b, ASP and the hydrolysates all exhibited typical protein absorption bands near the amide A band (3500–3200 cm^−1^, N–H stretching vibration [51]), amide I band (1700–1600 cm^−1^, mainly assigned to C=O stretching vibration [52]), and amide II band (1550–1530 cm^−1^, N–H bending and C–N stretching vibrations [53]). Compared with ASP, the amide I bands of the hydrolysates showed only slight changes in peak position rather than a pronounced shift toward lower wavenumbers. The amide I band of ASP appeared at 1654.14 cm^−1^, whereas the corresponding bands of SPH-Pap, SPH-Alc, and SPH-opt shifted to 1653.66, 1651.73, and 1652.21 cm^−1^, respectively. These slight changes indicate that enzymatic hydrolysis modified the hydrogen-bonding environment and secondary structure of the protein, but did not cause a pronounced displacement of the main amide I band [54].

To further compare secondary-structure changes, the amide I band was subjected to Gaussian fitting (Figure 4c–f). The results showed that β-turns and α-helices accounted for relatively high proportions in ASP, with values of 37.20% and 23.26%, respectively, indicating that the raw protein retained a considerable amount of ordered structure. After enzymatic hydrolysis, the secondary-structure proportions of the hydrolysates were redistributed [55]. In SPH-opt, the β-turn content decreased to 19.70%, whereas the random coil and β-sheet contents increased to 25.15% and 30.19%, respectively. These results indicate that sequential hydrolysis redistributed the fitted secondary-structure components and produced a pattern distinct from ASP. However, FTIR deconvolution alone cannot establish that these structural differences caused the observed changes in dispersion or radical-scavenging capacity.

These secondary-structure changes should not be regarded as exclusive to sequential hydrolysis, because single-enzyme hydrolysis also induced structural rearrangement. However, compared with SPH-Pap and SPH-Alc, SPH-opt showed a different structural rearrangement pattern under the same total enzyme dosage and hydrolysis time, suggesting that the selected papain–alcalase sequence produced a distinct pattern of protein disassembly and peptide formation compared with the tested single-enzyme treatments.

#### 3.5.3. UV Absorption Characteristics of ASP and Its Hydrolysates

**Figure 5:**
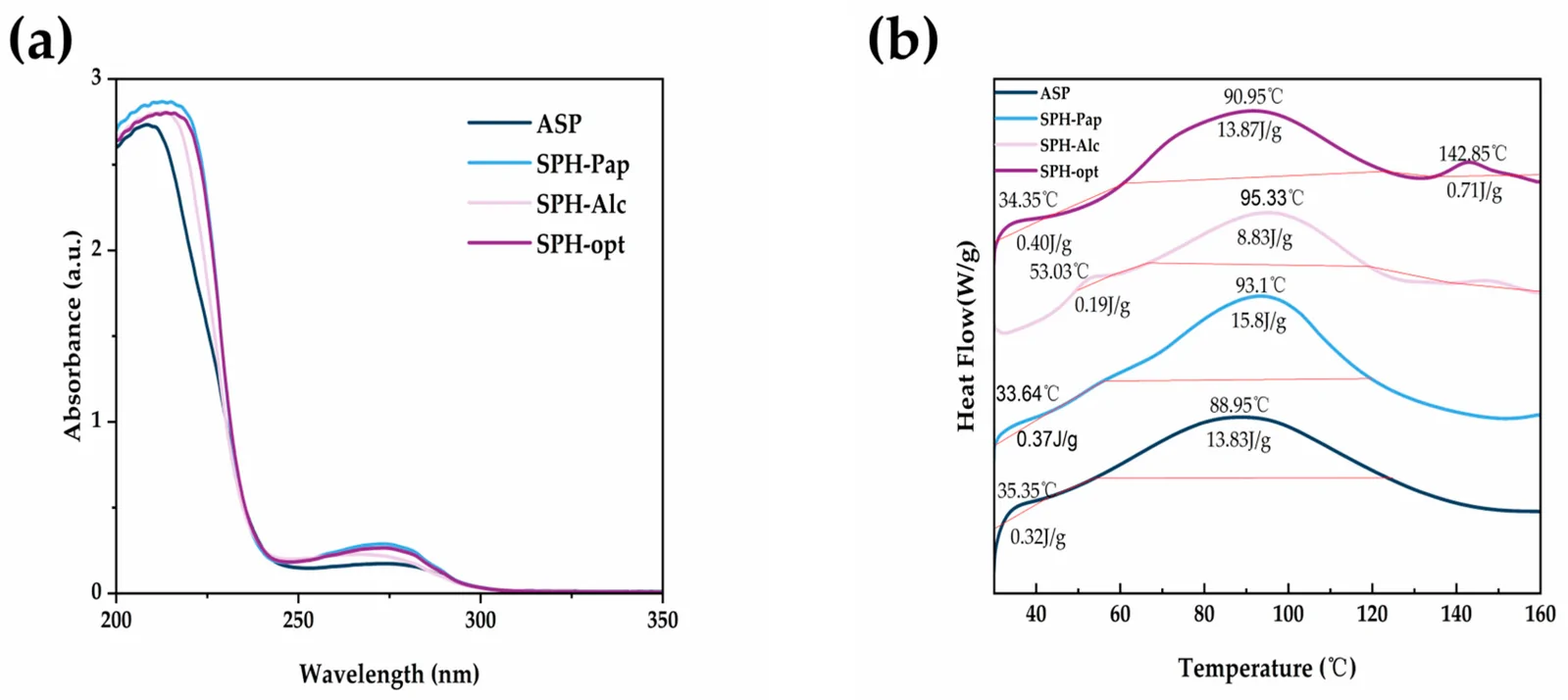
UV absorption spectra and DSC thermograms of ASP and its hydrolysates. (**a**) UV absorption spectra from 200 to 400 nm; (**b**) DSC thermograms. ASP, raw *Sthenoteuthis oualaniensis* protein; SPH-Pap, papain hydrolysate; SPH-Alc, alcalase hydrolysate; SPH-opt, optimized sequential hydrolysate. Representative profiles from three independent processing batches are shown.

UV spectroscopy provides indirect information on changes in peptide-backbone absorption and the microenvironment of aromatic amino acid residues during protein hydrolysis [56]. As shown in Figure 5a, ASP and the three hydrolysates exhibited characteristic absorption over the range of 200–400 nm. Compared with ASP, SPH-Pap, SPH-Alc, and SPH-opt showed increased absorbance near 280 nm, with stronger absorption observed for SPH-Pap and SPH-opt. These changes were consistent with alterations in the microenvironment and possible increased exposure of aromatic amino acid residues following enzymatic hydrolysis. However, changes in UV absorbance alone do not directly demonstrate protein unfolding or residue exposure.

Changes were also observed in the 220–240 nm region, particularly for SPH-Pap and SPH-opt. Because absorption in this region is associated mainly with peptide bonds and peptide-backbone structures, the observed changes were suggestive of hydrolysis-induced alterations in peptide-bond-related absorption and short-peptide formation. This interpretation was consistent with the SDS-PAGE and LC–MS/MS results, which showed degradation of high-molecular-weight components and differences in peptide profiles among the hydrolysates. Therefore, the UV spectra were interpreted as complementary evidence of hydrolysis-induced structural changes rather than as direct proof of specific backbone-cleavage or residue-exposure events.

#### 3.5.4. Thermal-Transition Behavior (DSC)

DSC was used to evaluate the effects of different enzymatic hydrolysis modes on the thermal-transition behavior of *S. oualaniensis* protein [57]. As shown in Figure 5b, ASP and the hydrolysates all exhibited thermal-transition peaks to varying extents. Enzymatic hydrolysis therefore did not completely eliminate the thermal response of the samples; instead, hydrolysis altered the thermal-transition behavior of the protein or peptide systems. The main thermal-transition peak of ASP was centered at approximately 88.95 °C. After hydrolysis, the main peak positions and peak shapes of SPH-Pap, SPH-Alc, and SPH-opt changed, indicating that protein-backbone cleavage, secondary-structure rearrangement, and inter-peptide interactions jointly affected the thermal response of the systems [58].

The main thermal-transition peak of SPH-opt was centered at approximately 90.95 °C, which was higher than that of ASP but lower than those of SPH-Pap and SPH-Alc. Sequential hydrolysis therefore did not simply increase or decrease thermal stability; rather, SPH-opt formed a peptide composition and aggregation state distinct from those of the single-enzyme hydrolysates. A weak high-temperature transition peak was also observed for SPH-opt, which may reflect a minor population of thermally stable components or aggregates. When considered together with the FTIR results, sequential hydrolysis appears to have induced both disassembly and reorganization of ASP. This structural state may be relevant to aqueous dispersibility and residue exposure, but the present data do not establish that it caused the relative radical-scavenging differences observed later [59].

#### 3.5.5. Peptidomic Analysis and Differences in Peptide Composition

**Figure 6:**
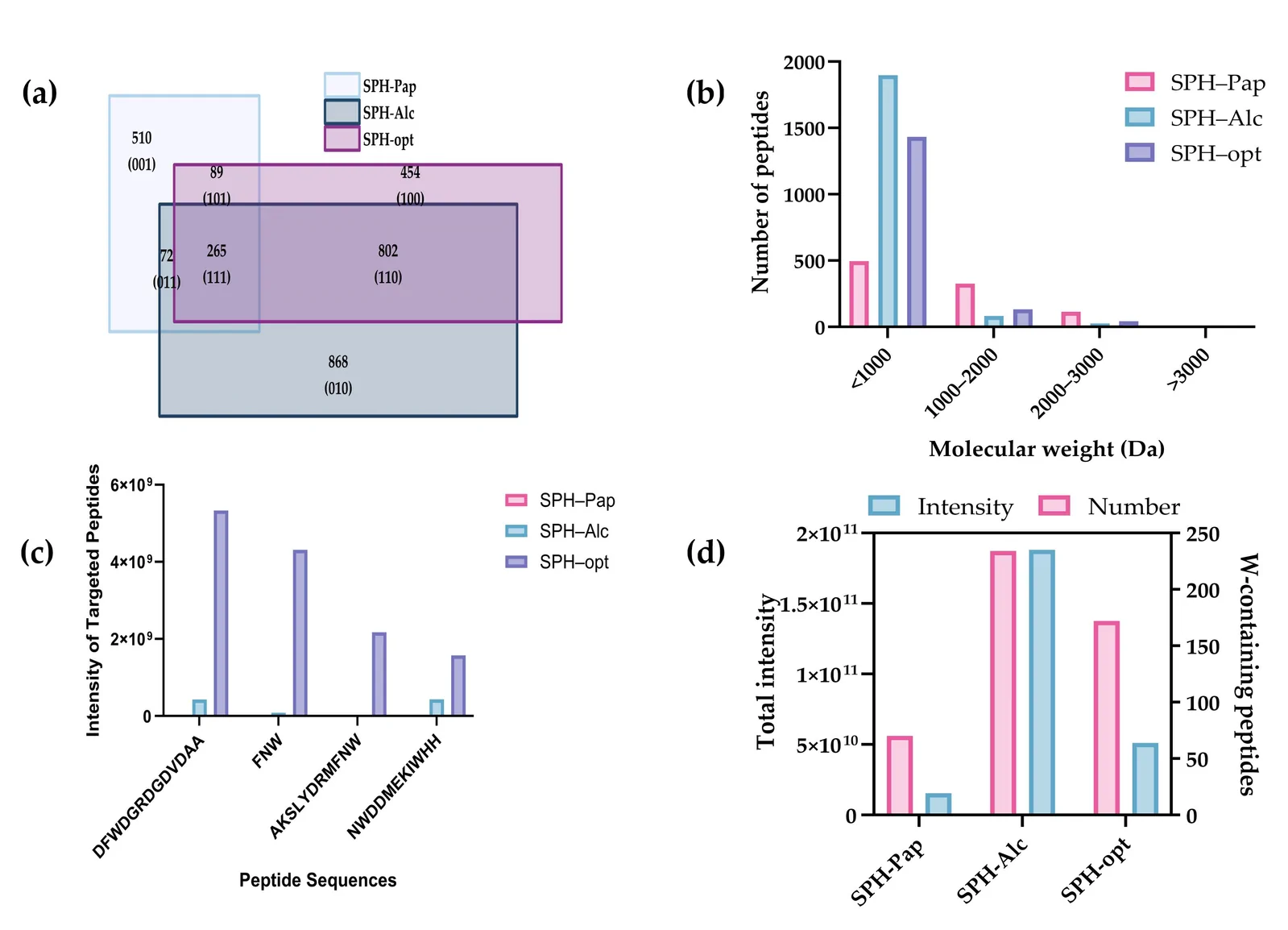
LC-MS/MS-based peptide profiling of SPH-Pap, SPH-Alc, and SPH-opt. (**a**) Shared and unique peptide distribution; (**b**) molecular weight distribution of identified peptides; (**c**) relative intensity of representative peptides; (**d**) total intensity and number of W-containing peptides.

To further compare the effects of different hydrolysis modes on peptide composition, SPH-Pap, SPH-Alc, and SPH-opt were analyzed by LC-MS/MS. As shown in Figure 6, peptides with different lengths and molecular-weight ranges were detected in all three hydrolysates, but the peptide-distribution patterns differed markedly. SPH-Pap retained a relatively large proportion of longer peptides, suggesting that single papain hydrolysis had a certain degree of selectivity but did not fully degrade some protein structures. SPH-Alc showed a higher number of short peptides and a higher total relative signal, which was consistent with more extensive cleavage under the present conditions. By comparison, the peptide distribution of SPH-opt was intermediate between those of the two single-enzyme hydrolysates, indicating a distinct peptide-length distribution and composition of characteristic peptides rather than a simple maximization of total peptide release.

Although SPH-Alc showed the highest number and total relative MS signal of Trp-containing peptides (Figure 6d), SPH-opt showed higher relative responses for several selected aromatic-residue-containing sequences (Table 5). FNW showed a 52.2-fold higher relative response in SPH-opt than in SPH-Alc, DFWDGRDGDVDAA showed a 12.5-fold higher response, and NWDDMEKIWHH showed a 3.64-fold higher response. AKSLYDRMFNW was detected in SPH-opt but not in SPH-Alc under the present LC-MS/MS conditions. None of these four representative sequences was detected in SPH-Pap under the same analytical conditions. Because peptides do not ionize equally in LC–MS/MS, the observed peptide-intensity values were interpreted as relative signals for comparative profiling rather than as absolute peptide concentrations [60]. The selected sequences should therefore be regarded as characteristic candidates associated with the SPH-opt profile, not as quantitatively proven functional components.

Mechanistically, papain pretreatment may weaken the aggregated myofibrillar structure and expose additional cleavage sites [12], whereas subsequent alcalase treatment may further cleave accessible protein backbones and alter the release pattern of short-to-medium peptides [13]. The DH and LC-MS/MS results together show that sequential hydrolysis did not globally maximize peptide-bond cleavage or the total number of aromatic-residue-containing peptides. Instead, it generated an intermediate DH and a distinct compositional profile in which a limited set of characteristic sequences displayed strongly higher relative responses. This distinction is important because peptide-level function depends on sequence context, residue position, charge, hydrophobicity, and chain length rather than on the presence of aromatic residues alone [38,39].

A sequence-specific comparison further shows why the four peptides should not be interpreted only from the presence of aromatic residues. FNW is a tripeptide containing Phe and Trp, so aromatic residues account for two of its three positions. AKSLYDRMFNW contains Tyr, Phe, and Trp together with Lys, Asp, and Arg, whereas NWDDMEKIWHH contains two Trp and two His residues in a sequence that also includes Asp, Glu, and Lys. DFWDGRDGDVDAA contains Phe and Trp but is dominated by multiple Asp residues. These contrasting combinations of aromatic, acidic, basic, and hydrophobic residues indicate that peptide length, residue position, and the surrounding sequence environment may jointly influence radical-scavenging behavior; the presence of Phe, Trp, or Tyr alone is insufficient to identify an antioxidant peptide [38,39].

Taken together, the LC-MS/MS results support a distinct peptide-composition profile in SPH-opt and identify sequences that merit follow-up, but none of the four peptides can be regarded as an experimentally confirmed antioxidant peptide from the present data. Because DPPH and ABTS are receptor-free chemical radical-scavenging assays, the present study does not define a relevant protein target for a mechanistically meaningful docking analysis. Quantitative peptidomics, purification or synthesis of the individual peptides, and direct activity testing are still required to establish peptide-specific contributions.

### 3.6. Analysis of Physical Properties

#### 3.6.1. Particle Size and Zeta Potential of ASP and Its Hydrolysates

**Figure 7:**
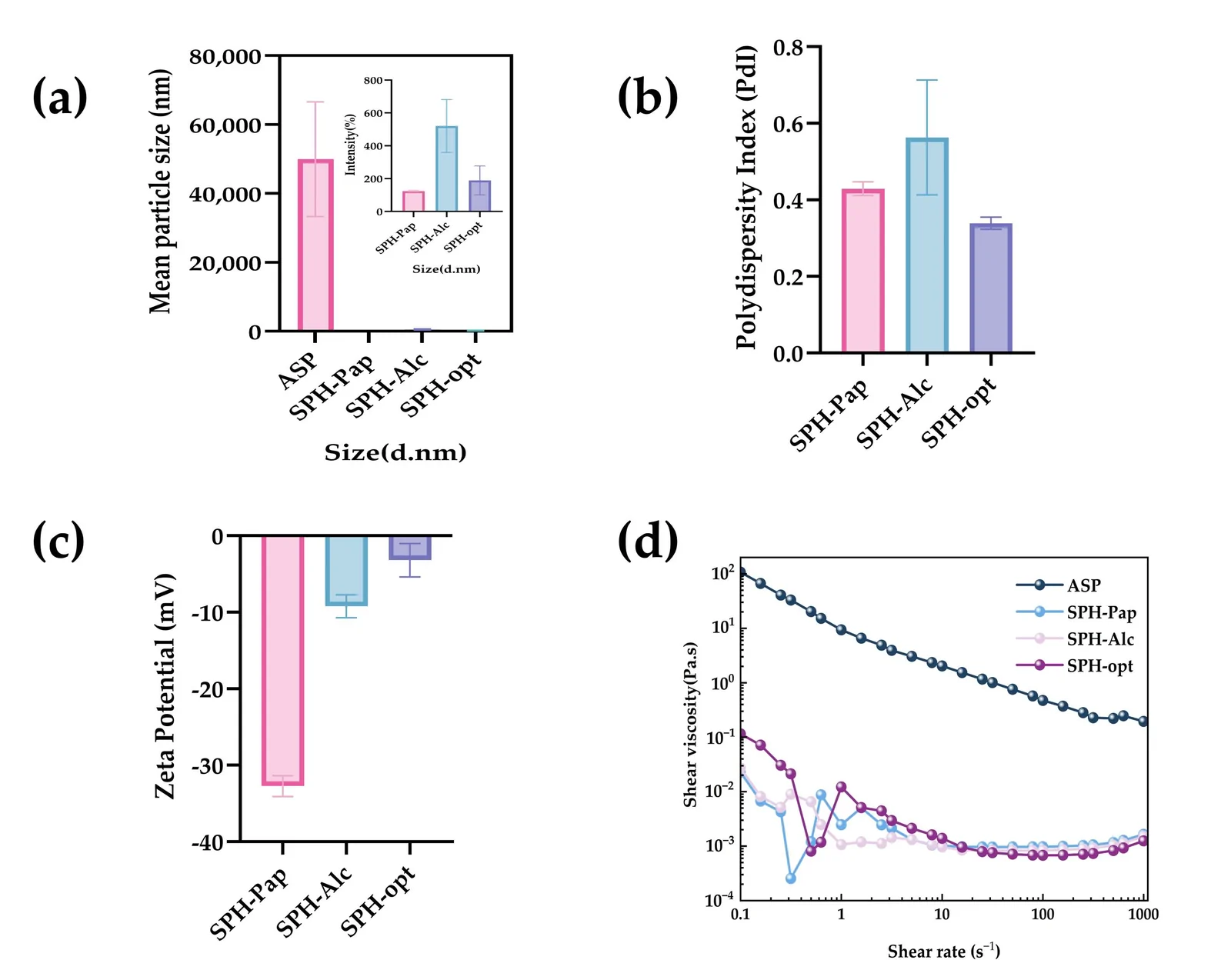
Particle characteristics and rheological behavior of ASP and its hydrolysates. (**a**) Mean particle size; (**b**) polydispersity index; (**c**) zeta potential; (**d**) apparent viscosity as a function of shear rate. Data in panels (**a**–**c**) are expressed as mean ± SD of three independent processing batches; panel (**d**) shows representative viscosity curves.

Particle size and zeta potential were used to evaluate the effects of enzymatic hydrolysis on protein particle dispersion and surface charge characteristics. As shown in Figure 7a–c, ASP had a mean particle size of approximately 50.0 µm, indicating that non-hydrolyzed *S. oualaniensis* protein existed mainly as large aggregates in the aqueous phase [61]. After hydrolysis, the particle sizes of all three hydrolysates decreased markedly to the nanoscale. The mean particle sizes of SPH-Pap, SPH-Alc, and SPH-opt were 124.4, 520.6, and 189.2 nm, respectively. These results indicate that enzymatic hydrolysis markedly reduced the apparent particle scale of the protein dispersions. Because ASP and the hydrolysates were measured using different particle-size methods, the particle-size data should mainly be used to indicate the transition from micrometre-scale particles to nanoscale particles, rather than for strictly equivalent absolute-value comparisons.

Different hydrolysis modes produced distinct effects on particle size. SPH-Pap showed the smallest particle size, at 124.4 nm, whereas SPH-opt had a particle size of 189.2 nm, markedly lower than that of SPH-Alc (520.6 nm). In addition, the PDI of SPH-opt was lower than that of SPH-Alc. These results suggest that sequential hydrolysis effectively reduced particle scale and produced smaller dispersed particles than single alcalase hydrolysis. This effect may be related to weakening of the protein aggregate structure by papain pretreatment, followed by further peptide-chain cleavage by alcalase, thereby reducing the amount of residual large particles.

The zeta potential results showed that SPH-Pap had a value of −32.73 mV, indicating strong electrostatic repulsion among particles. By contrast, the zeta potentials of SPH-Alc and SPH-opt were −9.21 and −3.20 mV, respectively, with relatively low absolute values; their dispersion stability may therefore not rely primarily on electrostatic repulsion. Considered together with the particle-size results, the dispersion behavior of SPH-opt may depend not only on electrostatic repulsion but also on peptide molecular-weight distribution, exposure of hydrophilic groups, and rebalanced interparticle interactions. Overall, sequential hydrolysis markedly reduced the particle scale of ASP. However, the low zeta potential of SPH-opt suggests that the dispersion-stabilization mechanism still requires further verification through storage-stability testing or microstructural observation.

#### 3.6.2. Rheological Properties (Viscosity)

Apparent viscosity reflects the flow behavior of sample dispersions under shear conditions. As shown in Figure 7d, ASP exhibited high viscosity at low shear rates, reaching 107.336 Pa·s at 0.1 s^−1^. As the shear rate increased, the viscosity decreased rapidly to 0.195 Pa·s at 1000 s^−1^, showing typical shear-thinning behavior [62]. This result is consistent with strong aggregation or network-like behavior of the raw protein dispersion under low-shear conditions.

After enzymatic hydrolysis, the viscosities of SPH-Pap, SPH-Alc, and SPH-opt were all markedly lower than that of ASP, indicating lower flow resistance after hydrolysis. The three hydrolysates also showed shear-thinning behavior, although their overall viscosities remained low. Among them, SPH-opt showed a relatively higher viscosity in the low-shear region, with a value of 0.115 Pa·s at 0.1 s^−1^, possibly due to weak interactions or transient aggregation among short peptides. Under medium-to-high shear rates, however, the viscosity of SPH-opt decreased rapidly and reached 0.00125 Pa·s at 1000 s^−1^, lower than those of SPH-Pap and SPH-Alc. These findings indicate that SPH-opt retained a measurable structural response under low-shear conditions but exhibited low flow resistance under high shear. Therefore, viscosity was not used as an independent functional endpoint in this study. Instead, it was included as a processing-related indicator to support the evaluation of hydrolysis-induced changes in dispersion and flow behavior. Together with particle size, zeta potential, and solubility, the viscosity results provide additional processing-related evidence that enzymatic hydrolysis altered the dispersion and flow behavior of *S. oualaniensis* protein hydrolysates in aqueous systems.

### 3.7. Analysis of Functional Properties

#### 3.7.1. pH-Dependent Solubility of ASP and Its Hydrolysates

**Figure 8:**
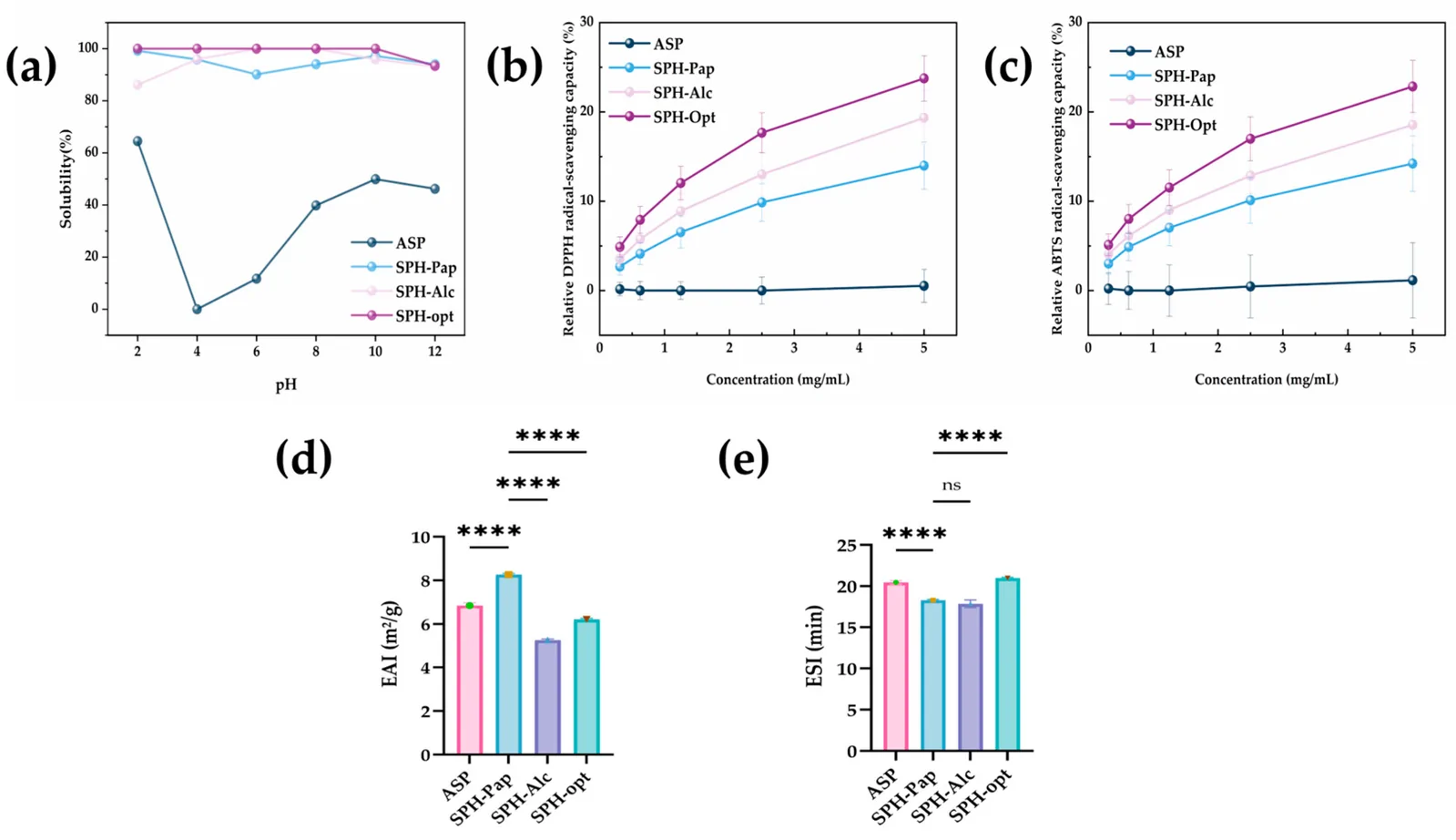
Functional properties of ASP and its hydrolysates. (**a**) Solubility at different pH values; (**b**) relative DPPH radical-scavenging capacity among the prepared samples; (**c**) relative ABTS radical-scavenging capacity among the prepared samples; (**d**) emulsifying activity index (EAI); (**e**) emulsion stability index (ESI). The DPPH and ABTS panels are intended only for within-study comparison because no external antioxidant standard was included. Data are presented as mean ± SD from three independent processing batches. Statistical significance annotations in panels (**d**,**e**) represent comparisons of each group with SPH-Pap only (**** *p* < 0.0001; ns, not significant).

Solubility is a key indicator for evaluating the processing suitability of protein hydrolysates [63]. As shown in Figure 8a, the solubility of ASP was strongly affected by pH. ASP was almost insoluble at pH 4.0, and the solubility was only 11.7% at pH 6.0, indicating that the raw protein readily underwent aggregation and precipitation under weakly acidic to near-neutral conditions.

After enzymatic hydrolysis, the solubilities of all three hydrolysates increased markedly. SPH-Pap maintained a high solubility of 90.1–99.2% over the pH range of 2.0–12.0. SPH-Alc reached 100% solubility at pH 6.0 and 8.0, but its solubility decreased to 86.1% under strongly acidic conditions. In comparison, SPH-opt maintained 100% solubility over pH 2.0–10.0 and still retained 93.4% solubility at pH 12.0. These results indicate that the selected sequential hydrolysis treatment broadened the pH range over which high solubility was retained under the tested conditions.

#### 3.7.2. Relative DPPH and ABTS Radical-Scavenging Capacities

The relative in vitro radical-scavenging capacities of the prepared samples were evaluated using DPPH and ABTS assays [64]. As shown in Figure 8b,c, ASP showed low scavenging rates, whereas the rates of the three hydrolysates increased with concentration. These chemical assays were used only to compare samples tested under identical conditions. Because no external antioxidant standard was included, the results cannot be interpreted as absolute antioxidant potency or directly compared with values expressed as Trolox, BHT, or ascorbic-acid equivalents.

At 5 mg/mL, the DPPH and ABTS scavenging rates of SPH-opt were 23.75% and 22.84%, respectively, and were moderately higher than those of SPH-Pap and SPH-Alc under the tested conditions. These responses were not RSM optimization variables: $A_{\mathrm{target}}$ was the process-optimization response, whereas DPPH and ABTS were measured afterward as comparative performance outcomes. Therefore, the co-occurrence of the highest $A_{\mathrm{target}}$ and the highest scavenging rates in SPH-opt does not demonstrate that optimizing $A_{\mathrm{target}}$ also optimized antioxidant performance, nor does it establish a causal relationship between the two. The absolute scavenging rates were moderate, and SPH-opt should not be described as a highly potent antioxidant hydrolysate.

DPPH and ABTS are chemical radical-scavenging assays and provide only preliminary comparative evidence under the present in vitro conditions. The absence of a positive standard precludes potency benchmarking, and IC_50_ values were not determined. The LC-MS/MS profiling and sequence-specific comparison identify structurally interesting candidates but do not verify individual peptide activity. Future work should include appropriate positive controls, concentration ranges suitable for IC_50_ determination, activity-guided fractionation, peptide synthesis or purification, and validation in cellular or food-matrix systems.

#### 3.7.3. Emulsifying Activity and Emulsion Stability

The emulsifying activity index (EAI) reflects the ability of a sample to form an oil–water interface, whereas the emulsion stability index (ESI) reflects the short-term stability of the resulting emulsion [65]. As shown in Figure 8d, SPH-Pap exhibited the highest EAI (8.26 m^2^/g) and was significantly higher than ASP, SPH-Alc, and SPH-opt (*p* < 0.0001 for all comparisons against SPH-Pap). SPH-opt exhibited an EAI of 6.21 m^2^/g, which was significantly lower than that of SPH-Pap, numerically lower than that of ASP, and numerically higher than that of SPH-Alc. Therefore, the selected sequential hydrolysis treatment did not improve initial emulsifying activity relative to papain hydrolysis. Extensive peptide-bond cleavage and the formation of shorter peptides may reduce peptide adsorption and cohesive-film formation at the oil–water interface [66].

As shown in Figure 8e, SPH-opt exhibited the numerically highest ESI (20.98 min), followed by ASP (20.45 min), SPH-Pap (18.30 min), and SPH-Alc (17.87 min). Compared with SPH-Pap, the ESI values of ASP and SPH-opt were significantly higher (*p* < 0.0001), whereas SPH-Alc did not differ significantly from SPH-Pap. Because the statistical annotations in Figure 8e represent comparisons against SPH-Pap only, the differences between SPH-opt and ASP or SPH-Alc are described as numerical rather than statistically significant. Thus, SPH-opt showed favorable short-term emulsion stability under the tested conditions, although its ESI was only numerically slightly higher than that of ASP.

Because interfacial tension, adsorption kinetics, and emulsion microstructure were not measured, the mechanism underlying the difference between EAI and ESI remains preliminary. Further direct interfacial measurements and validation in specific food matrices are required.

## 4. Conclusions

In this study, the GFC target-window peak area ($A_{\mathrm{target}}$) was used as a peptide-distribution-oriented operational index to optimize the papain–alcalase sequential hydrolysis of *Sthenoteuthis oualaniensis* protein. Under the preferred conditions of 1110 U/g dry ASP, a papain/alcalase mass ratio of 3:5, and a total hydrolysis time of 8 h, $A_{\mathrm{target}}$ reached 0.0648 ± 0.0012 a.u. SPH-Alc showed the highest OPA-derived DH, whereas SPH-opt showed the highest $A_{\mathrm{target}}$, indicating that DH and $A_{\mathrm{target}}$ provide complementary information on overall peptide-bond cleavage and peptide distribution within the selected GFC window.

Compared with the tested single-enzyme hydrolysates, SPH-opt showed limited residual high-molecular-weight components, high solubility across the tested pH range, a distinct LC–MS/MS peptide profile, and the numerically highest ESI. However, the functional properties were evaluated after process optimization and were not included as RSM response variables. Moreover, because no external antioxidant standard or IC_50_ determination was included, the radical-scavenging results support only relative within-study comparisons. Therefore, the present results do not establish causal relationships among $A_{\mathrm{target}}$, peptide composition, and functional performance.

The principal limitations of this study include the estimation of protein-equivalent contents from amino acid composition, the absence of reverse-order and simultaneous dual-enzyme controls, and the lack of quantitative peptidomics and direct verification of individual peptide activities. Future studies should include total-nitrogen-based protein normalization, quantitative peptidomics, activity-guided fractionation, direct testing of purified or synthesized peptides, and validation using cellular and food-matrix systems. Interfacial properties and emulsion microstructure should also be examined to clarify the observed differences between EAI and ESI.

## Figures and Tables

**Table 1:** Independent variables and coded levels used in the Box–Behnken design.

Level	A: Total Enzyme Dosage (U/g Dry ASP)	B: Papain/Alcalase Ratio (%)	C: Hydrolysis Time (h)
−1	980	25.0	6
0	1120	37.5	8
1	1260	50.0	10

Note: B represents the percentage of papain mass in the total mass of the two added enzymes. The corresponding papain/alcalase mass ratios were 1:3, 3:5, and 1:1, respectively.

**Table 2:** Amino acid composition and nutritional indices of ASP and its hydrolysates.

Amino Acid /Index	ASP (g/100 g)	SPH-Pap (g/100 g)	SPH-Alc (g/100 g)	SPH-opt (g/100 g)	FAO/WHO Pattern (g/100 g)
Aspartic acid	8.29 ± 0.12	8.08 ± 0.10	7.74 ± 0.54	8.12 ± 0.04	-
Threonine	3.55 ± 0.05	3.42 ± 0.04	3.59 ± 0.16	3.75 ± 0.09	2.3
Serine	4.02 ± 0.05	4.14 ± 0.04	4.07 ± 0.20	4.31 ± 0.05	-
Glutamic acid	14.18 ± 0.19	13.91 ± 0.09	12.83 ± 0.82	13.06 ± 0.13	-
Glycine	4.96 ± 0.06	5.86 ± 0.07	5.16 ± 0.32	5.75 ± 0.06	-
Alanine	6.27 ± 0.08	6.64 ± 0.08	6.07 ± 0.39	6.45 ± 0.07	-
Cysteine	0.15 ± 0.01	0.16 ± 0.00	0.15 ± 0.01	0.17 ± 0.01	See Met
Valine	3.65 ± 0.05	3.50 ± 0.04	3.32 ± 0.19	3.44 ± 0.03	3.9
Methionine	0.53 ± 0.01	0.35 ± 0.00	0.65 ± 0.04	0.50 ± 0.01	2.2, as Met + Cys
Isoleucine	3.20 ± 0.04	2.92 ± 0.03	2.87 ± 0.14	3.01 ± 0.02	3.0
Leucine	8.35 ± 0.11	8.06 ± 0.09	7.55 ± 0.44	8.16 ± 0.08	5.9
Tyrosine	2.50 ± 0.03	2.45 ± 0.03	2.76 ± 0.15	2.72 ± 0.02	See Phe
Phenylalanine	3.12 ± 0.04	2.97 ± 0.04	2.94 ± 0.16	3.12 ± 0.03	3.8, as Phe + Tyr
Lysine	7.42 ± 0.10	8.33 ± 0.10	6.84 ± 0.44	7.21 ± 0.07	4.5
Histidine	1.93 ± 0.02	1.76 ± 0.02	1.69 ± 0.09	1.63 ± 0.01	1.5
Arginine	6.23 ± 0.08	6.78 ± 0.08	5.89 ± 0.32	6.40 ± 0.06	-
Proline	2.65 ± 0.04	2.67 ± 0.04	2.77 ± 0.12	2.80 ± 0.03	-
Total amino acids	81.00	82.00	76.89	80.60	-
EAA/TAA (%)	39.20	38.18	38.30	38.24	≥27.1
EAA/NEAA (%)	64.47	61.77	62.08	61.91	-
HAA/TAA (%)	37.37	36.05	37.63	37.47	-

Note: In the FAO/WHO pattern, the recommended values for sulfur-containing and aromatic amino acids correspond to Met + Cys and Phe + Tyr, respectively. TAA, total amino acids; EAA, essential amino acids, including Thr, Val, Met, Ile, Leu, Phe, Lys, and His; NEAA, non-essential amino acids; HAA, hydrophobic amino acids, including Ala, Val, Ile, Leu, Tyr, Phe, Pro, and Met. Trp was not detected under the analytical conditions used in this study; therefore, Trp was not listed in the table or included in the calculation of related ratios. Values are expressed as mean ± SD of three independent processing batches.

**Table 4:** Comparison of OPA-derived degree of hydrolysis and GFC target-window peak area of the comparative *S. oualaniensis* protein hydrolysates.

Sample	DH (%)	GFC Target-Window Peak Area ($A_{\mathrm{target}}$, a.u.)
SPH-Pap	10.72 ± 0.08	0.0528 ± 0.0015
SPH-Alc	35.32 ± 0.12	0.0485 ± 0.0018
SPH-opt	25.03 ± 0.37	0.0648 ± 0.0012

Note: Values are expressed as mean ± SD of three independent processing batches. All pairwise differences within each column were significant at *p* < 0.05. The protein-equivalent percentage used for DH calculation was estimated from the measured amino acid composition.

**Table 5:** Representative peptides showing higher relative LC-MS/MS responses in SPH-opt.

Peptide Sequence	Aromatic Residues	Putative Source Protein	SPH-opt Relative MS Intensity (a.u.)	SPH-Alc Relative MS Intensity (a.u.)	Opt/Alc Ratio
FNW	F, W	Myosin-related protein	4.32 × 10^9^	8.28 × 10^7^	52.2
AKSLYDRMFNW	Y, F, W	Myosin heavy chain-related protein	2.18 × 10^9^	ND	Opt-specific
NWDDMEKIWHH	W (2)	Actin I	1.58 × 10^9^	4.33 × 10^8^	3.64
DFWDGRDGDVDAA	F, W	Myosin catalytic light chain LC-1	5.34 × 10^9^	4.27 × 10^8^	12.5

Note: Representative peptides were selected according to their higher relative responses in SPH-opt. Source proteins were assigned by database matching and should be regarded as putative origins. Intensities are comparative MS signals rather than absolute peptide concentrations. ND, not detected under the present LC-MS/MS conditions; all four representative sequences were ND in SPH-Pap. Fold ratios were calculated directly from the exported peptide-intensity values and only when the peptide was detected in both SPH-opt and SPH-Alc.

## Data Availability

The original contributions presented in this study are included in the article/supplementary material. Further inquiries can be directed to the corresponding authors.

## Supplementary Materials

The following supporting information can be downloaded at https://www.mdpi.com/article/10.3390/foods15172998/s1. Table S1: Analysis of variance (ANOVA) for the fitted quadratic model of the GFC target-window peak area.

## References

[1] Li N., Diao X., Pu X., Tang P., Elango J., Wu W. (2023). The antioxidant protective effect of iris-squid-derived protein hydrolysates (>10 kDa) in HSF fibroblast cells induced by H_2_O_2_. J. Compos. Sci..

[2] Wang J., Xu Z., Lu W., Zhou X., Liu S., Zhu S., Ding Y. (2024). Improving the texture attributes of squid meat (*Sthenoteuthis oualaniensis*) with slight oxidative and phosphate curing treatments. Food Res. Int..

[3] Lee S., Hyun J., Lim J., Jeon Y.-J., Ryu B. (2026). Value-added utilization of undersized *Paralichthys olivaceus* through enzymatic hydrolysis: Fish protein supports recovery of muscle strength in age-related muscle loss. Food Res. Int..

[4] Coscueta E.R., Cunha Fernandes N., Brassesco M.E., Rosa A., Almeida A., Pintado M.M. (2024). Turning discarded blue shark (*Prionace glauca*) skin into a valuable nutraceutical resource: An enzymatic collagen hydrolysate. Food Biosci..

[5] Fuentes C., Verdú S., Grau R., Barat J.M., Fuentes A. (2025). In vitro antioxidant and antidiabetic effects of Atlantic mackerel and sardine by-product hydrolysates. Mar. Drugs.

[6] Du M., Yu W., Ding N., Jian M., Cheng Y., Gan J. (2024). Antioxidant, aroma, and sensory characteristics of Maillard reaction products from *Urechis unicinctus* hydrolysates: Development of food flavorings. Front. Nutr..

[7] Ramakrishnan S.R., Jeong C.-R., Park J.-W., Cho S.-S., Kim S.-J. (2023). A review on the processing of functional proteins or peptides derived from fish by-products and their industrial applications. Heliyon.

[8] Nikoo M., Regenstein J.M., Yasemi M. (2023). Protein hydrolysates from fishery processing by-products: Production, characteristics, food applications, and challenges. Foods.

[9] Zeng J., Zou J., Zhao J., Lin K., Zhang L., Yi H., Gong P. (2023). Chymosin pretreatment accelerated papain catalysed hydrolysis for decreasing casein antigenicity by exposing the cleavage site at tyrosine residues. Food Chem..

[10] Hu X., Yang Y., Chang C., Li J., Su Y., Gu L. (2024). The targeted development of collagen-active peptides based on composite enzyme hydrolysis: A study on the structure–activity relationship. Food Funct..

[11] Moreno-Mariscal C., Moroni F., Pérez-Sánchez J., Mora L., Toldrá F. (2025). Optimization of sequential enzymatic hydrolysis in porcine blood and the influence on peptide profile and bioactivity of prepared hydrolysates. Int. J. Mol. Sci..

[12] Tacias-Pascacio V.G., Castañeda-Valbuena D., Morellon-Sterling R., Tavano O., Berenguer-Murcia Á., Vela-Gutiérrez G., Rather I.A., Fernandez-Lafuente R. (2021). Bioactive peptides from fisheries residues: A review of use of papain in proteolysis reactions. Int. J. Biol. Macromol..

[13] Petushkova A.I., Savvateeva L.V., Zamyatnin A.A. (2022). Structure determinants defining the specificity of papain-like cysteine proteases. Comput. Struct. Biotechnol. J..

[14] Hao L., Li X., Zhao B., Song X., Zhang Y., Liang Q. (2024). Enzymatic hydrolysis optimization of yak whey protein concentrates and bioactivity evaluation of the ultrafiltered peptide fractions. Molecules.

[15] Rios-Morales S.N., Brito-de la Fuente E., Aguilar-Uscanga M.G., Torrestiana-Sánchez B. (2023). Optimization of egg yolk protein hydrolysis by RSM and properties of hydrolysates. J. Appl. Res. Technol..

[16] Liu Z., Wang S., Cao R., Hu M. (2025). Review on bioactive peptides from Antarctic krill: From preparation to structure–activity relationship and tech-functionality. Curr. Res. Food Sci..

[17] Lu J., Shi P., Cao Y., Shi B., Shen H., Zhao S., Gao Y., Chi H., Wang L., Shi Y. (2025). Isolation and purification of novel antioxidant peptides from mussel (*Mytilus edulis*) prepared by marine *Bacillus velezensis* Z-1 protease. Mar. Drugs.

[18] Xia X., Song S., Zhou T., Zhang H., Cui H., Zhang F., Hayat K., Zhang X., Ho C.-T. (2023). Preparation of saltiness-enhancing enzymatic hydrolyzed pea protein and identification of the functional small peptides of salt reduction. J. Agric. Food Chem..

[19] Abdo A.A.A., Al-Dalali S., Hou Y., Aleryani H., Shehzad Q., Asawmahi O., AL-Farga A., Mohammed B., Liu X., Sang Y. (2024). Modification of marine bioactive peptides: Strategy to improve the biological activity, stability, and taste properties. Food Bioprocess Technol..

[20] Li S., Gu J., Zhong B., Feng R., Pan H., Liu Y., Shi W. (2025). Isolation and purification of antioxidant peptides from swim bladder of grass carp (*Ctenopharyngodon idella*). Aquac. Fish..

[21] Moghadam M., Heyn T.R., Schwarz K., Keppler J.K. (2024). Influence of purification on the composition, structural and physicochemical properties of myofibrillar proteins from blue mussel (*Mytilus edulis*). Food Biosci..

[22] D’Atri V., Imiołek M., Quinn C., Finny A., Lauber M., Fekete S., Guillarme D. (2024). Size exclusion chromatography of biopharmaceutical products: From current practices for proteins to emerging trends for viral vectors, nucleic acids and lipid nanoparticles. J. Chromatogr. A.

[23] Cernosek T., Jain N., Dalphin M., Behrens S., Wunderli P. (2024). Accelerated development of a SEC-HPLC procedure for purity analysis of monoclonal antibodies using design of experiments. J. Chromatogr. B.

[24] Nielsen P.M., Petersen D., Dambmann C. (2001). Improved method for determining food protein degree of hydrolysis. J. Food Sci..

[25] Arias-Moscoso J.L., Maldonado-Arce A., Rouzaud-Sández O., Márquez-Ríos E., Torres-Arreola W., Santacruz-Ortega H., Gaxiola-Cortés M.G., Ezquerra-Brauer J.M. (2015). Physicochemical characterization of protein hydrolysates produced by autolysis of jumbo squid (*Dosidicus gigas*) byproducts. Food Biophys..

[26] Akbarbaglu Z., Ayaseh A., Ghanbarzadeh B., Sarabandi K. (2022). Techno-functional, biological and structural properties of Spirulina platensis peptides from different proteases. Algal Res..

[27] Schägger H. (2006). Tricine–SDS-PAGE. Nat. Protoc..

[28] Luo J., Zhang M., Zeng Y., Guo H., Wu X., Meng Z., Yin R. (2024). Structural and functional properties of protein hydrolysates from myofibrillar protein of crocodile (*Crocodylus siamensis*) meat. LWT.

[29] Wang H., Zhao S., Xia X., Liu J., Sun F., Kong B. (2024). Interaction of the extracellular protease from *Staphylococcus xylosus* with meat proteins elucidated via spectroscopic and molecular docking. Food Chem. X.

[30] Khan M.U., Hamid K., Tolstorebrov I., Rustad T., Eikevik T.M., Watanabe M. (2025). Evaluation of Atlantic cod hydrolysate properties in innovative freeze concentration techniques. Food Chem. X.

[31] Sun X., Tan Y., Duan C., Wang Q., Cui Q. (2023). Effects of processing on component interactions and peptide profiles of preterm infant formula based on peptidomics by Q Exactive plus analysis. LWT.

[32] Zhu B., Yang J., Yu J., Dou J., Ning Y., Qi B., Li Y. (2024). Effects of L-arginine/L-lysine modifications on the protein structure, binding interactions, and functional properties of soy protein hydrolysate. Food Hydrocoll..

[33] Chen X., Yi J., Wen Z., Fan Y. (2024). Ultrasonic pretreatment and epigallocatechin gallate incorporation enhance the formation, apparent viscosity, and antioxidant activity of pea protein amyloid-like fibrils. Food Hydrocoll..

[34] Huang D., Xu Y., Zhang W., Liu Y., Zhang T., Liu H., Jiang Y., Li D. (2024). Enhancement of foaming property of ormosia protein: Insights into the effect of high-intensity ultrasound on physicochemical properties and structure analysis. Food Hydrocoll..

[35] Dong Y., Yan W., Zhang Y.-Q. (2022). Effects of spray drying and freeze drying on physicochemical properties, antioxidant and ACE inhibitory activities of bighead carp (*Aristichthys nobilis*) skin hydrolysates. Foods.

[36] Yang Y., Sun S., Zou L., Wang B., Bian X., Zhu P., Ren L., Shi Y., Zhang N. (2022). Characterization of structural and functional properties of soybean 11S globulin during the renaturation after the guanidine hydrochloride denaturation. Food Hydrocoll..

[37] FAO, WHO, UNU (2007). Protein and Amino Acid Requirements in Human Nutrition: Report of a Joint FAO/WHO/UNU Expert Consultation.

[38] Mirzaee H., Ahmadi Gavlighi H., Nikoo M., Udenigwe C.C., Khodaiyan F. (2023). Relation of amino acid composition, hydrophobicity, and molecular weight with antidiabetic, antihypertensive, and antioxidant properties of mixtures of corn gluten and soy protein hydrolysates. Food Sci. Nutr..

[39] Zheng L., Zhao Y., Dong H., Su G., Zhao M. (2016). Structure–activity relationship of antioxidant dipeptides: Dominant role of Tyr, Trp, Cys and Met residues. J. Funct. Foods.

[40] Le Maux S., Nongonierma A.B., Barre C., FitzGerald R.J. (2016). Enzymatic generation of whey protein hydrolysates under pH-controlled and non-pH-controlled conditions: Impact on physicochemical and bioactive properties. Food Chem..

[41] Wang Y., Song Y., Chang Y., Liu Y., Chen G., Xue C. (2022). Dynamic changes of peptidome and release of polysaccharide in sea cucumber (*Apostichopus japonicus*) hydrolysates depending on enzymatic hydrolysis approaches. Food Sci. Hum. Wellness.

[42] Garmidolova A., Desseva I., Mihaylova D., Fidan H., Terziyska M., Pavlov A. (2022). Papain hydrolysates of lupin proteins with antioxidant, antimicrobial, and acetylcholinesterase inhibitory activities. Appl. Sci..

[43] Nunez S.M., Valencia P., Solis T., Valdivia S., Cardenas C., Guzman F., Astuya A., Romero J., Jaques A., Gauthier S.F. (2024). Enzymatic hydrolysis of salmon frame proteins using a sequential batch operational strategy: An improvement in water-holding capacity. Foods.

[44] Lopez-Martinez M.I., Toldra F., Mora L. (2024). Sequential enzymatic hydrolysis and ultrasound pretreatment of pork liver for the generation of bioactive and taste-related hydrolyzates. J. Agric. Food Chem..

[45] Begum N., Khan Q.U., Al-Dalali S., Lu D., Yang F., Li J., Ma M., Li C., Cai Z. (2024). Process optimization and identification of antioxidant peptides from enzymatic hydrolysate of bovine bone extract, a potential source in cultured meat. Front. Sustain. Food Syst..

[46] Matic J., Borilden B., Sorokina L., Ronning S.B., Kristoffersen K.A., Afseth N.K., Dankel K., Wubshet S.G. (2025). Facilitated identification of bioactive peptide fractions and optimization of enzymatic protein hydrolysis using size-exclusion chromatography fingerprints: Combining interval PLS and response surface modeling. Talanta.

[47] Qi Q., Zhang G., Wang W., Sadiq F.A., Zhang Y., Li X., Chen Q., Xia Q., Wang X., Li Y. (2022). Preparation and antioxidant properties of germinated soybean protein hydrolysates. Front. Nutr..

[48] Rao P.S., Bajaj R., Mann B. (2020). Impact of sequential enzymatic hydrolysis on antioxidant activity and peptide profile of casein hydrolysate. J. Food Sci. Technol..

[49] Liang Y., Guo Y., Zheng Y., Liu S., Cheng T., Zhou L., Guo Z. (2022). Effects of high-pressure homogenization on physicochemical and functional properties of enzymatic hydrolyzed soybean protein concentrate. Front. Nutr..

[50] Ramírez-Suárez J.C., Álvarez-Armenta A., Mazorra-Manzano M.Á., Scheuren Acevedo S.M., Pacheco-Aguilar R., García-Sánchez G., Carvallo M.G., Ramírez-Guerra H.E. (2024). Cryoprotective effect of low molecular-mass nitrogen compounds on the myofibrillar protein of the jumbo squid (*Dosidicus gigas*) muscle. Food Sci. Technol..

[51] Śmiszek-Lindert W.E., Chełmecka E., Góralczyk S., Kaczmarek M. (2017). Vibrational spectroscopic (FT-IR, FT-Raman) studies, Hirshfeld surfaces analysis, and quantum chemical calculations of m-acetotoluidide and m-thioacetotoluidide. J. Mol. Struct..

[52] Lyu S., Chen M., Wang Y., Zhang D., Zhao S., Liu J., Pan F., Zhang T. (2023). Foaming properties of egg white proteins improved by enzymatic hydrolysis: The changes in structure and physicochemical properties. Food Hydrocoll..

[53] Kupser P., Pagel K., Oomens J., Polfer N., Koksch B., Meijer G., von Helden G. (2010). Amide-I and -II vibrations of the cyclic β-sheet model peptide gramicidin S in the gas phase. J. Am. Chem. Soc..

[54] Alahmad K., Noman A., Xia W., Jiang Q., Xu Y. (2023). Influence of the enzymatic hydrolysis using Flavourzyme enzyme on functional, secondary structure, and antioxidant characteristics of protein hydrolysates produced from bighead carp (*Hypophthalmichthys nobilis*). Molecules.

[55] Oliveira L.C., Martinez-Villaluenga C., Frias J., Cartea M.E., Francisco M., Cristianini M., Peñas E. (2024). High pressure-assisted enzymatic hydrolysis potentiates the production of quinoa protein hydrolysates with antioxidant and ACE-inhibitory activities. Food Chem..

[56] Wei M., Ning C., Ren Y., Hu F., Wang M., Li W. (2024). Characterisation and comparison of enzymatically prepared donkey milk whey protein hydrolysates. Food Chem. X.

[57] Rout P., Chakraborty C., Hossain S. (2024). Functional characterization of enzyme-hydrolysed soy and whey protein isolates: A comparative approach. Food Chem. Adv..

[58] Dent T., Campanella O., Maleky F. (2023). Enzymatic hydrolysis of soy and chickpea protein with Alcalase and Flavourzyme and formation of hydrogen bond mediated insoluble aggregates. Curr. Res. Food Sci..

[59] Qoms M.S., Arulrajah B., Shamsudin R., Ramli N.S., Ibadullah W.Z.W., Chau D.-M., Saari N. (2023). Enzymolysis of *Azolla pinnata* protein concentrate: Effect of protease types and hydrolysis extents on the physicochemical, techno-functional and biological properties. Food Biosci..

[60] Gunter H.M., Youlten S.E., Reis A.L.M., McCubbin T., Madala B.S., Wong T., Stevanovski I., Cipponi A., Deveson I.W., Santini N.S. (2024). A universal molecular control for DNA, mRNA and protein expression. Nat. Commun..

[61] Akbarbaglu Z., Sarabandi K., Peighambardoust S.H., Sarabandi R., Kafil H.S., Hesarinejad M.A. (2024). Enzymatic modification of cold pressed coconut meal protein: Nutritional, functional and biological properties. Sustain. Food Technol..

[62] Queirós R.P., Moreira N., Pinto C.A., Fidalgo L.G., Saraiva J.A., Lopes-da-Silva J.A. (2024). Influence of high-pressure processing and microbial transglutaminase on the properties of pea protein isolates. Macromol.

[63] Barea P., Melgosa R., Benito-Roman O., Illera A.E., Beltran S., Sanz M.T. (2024). Green fractionation and hydrolysis of fish meal to improve their techno-functional properties. Food Chem..

[64] Wang L., Li Z., Fan X., Zhang T., Wang H., Ye K. (2024). Novel antioxidant peptides from bovine blood: Purification, identification and mechanism of action. LWT.

[65] Zhao X., Zheng H., Sun Y., Zhang M., Geng M., Li Y., Teng F. (2022). Effect of enzymatic hydrolysis conditions on structure of soy protein isolate/gum arabic complex and stability of oil-in-water emulsion. J. Sci. Food Agric..

[66] Gregersen Echers S., Jafarpour A., Yesiltas B., García-Moreno P.J., Greve-Poulsen M., Hansen D.K., Jacobsen C., Overgaard M.T., Hansen E.B. (2023). Targeted hydrolysis of native potato protein: A novel workflow for obtaining hydrolysates with improved interfacial properties. Food Hydrocoll..

