# Supplementary material for: Gel Filtration Chromatography-Guided Sequential Hydrolysis of Sthenoteuthis oualaniensis Protein: Peptide Distribution and Functional Properties
Source: Foods. 2026 Aug 26;15(17):2998. doi: 10.3390/foods15172998 (PMC13565722; doi:10.3390/foods15172998)
Supplement: Supplementary file 1 [file foods-15-02998-s001.zip › foods-4474547-supplementary.pdf]

Supplementary Materials

Table S1. Analysis of variance (ANOVA) for the fitted quadratic model of the GFC target-window peak area.

**Source**	**Sum of squares ($\times 10^{-4}$)**	**df**	**Mean square ($\times 10^{-4}$)**	**F-value**	**p-value**	**Significance**
Model	15	9	1.67	43.67	< 0.0001	**
A	0.28	1	0.28	7.47	0.0292	*
B	0.01	1	0.01	0.16	0.6985	
C	0.01	1	0.01	0.37	0.564	
AB	0.09	1	0.09	2.34	0.1697	
AC	0.14	1	0.14	3.69	0.0963	
BC	0.06	1	0.06	1.49	0.2622	
A^2	4.55	1	4.55	122.52	< 0.0001	**
B^2	5.44	1	5.44	146.58	< 0.0001	**
C^2	2.59	1	2.59	69.78	< 0.0001	**
Residual	0.26	7	0.04			
Lack of fit	0.02	3	0.01	0.12	0.9415	
Pure error	0.24	4	0.06			
Total	15.26	16				

Note: * $p < 0.05$; ** $p < 0.01$. A, total enzyme dosage; B, papain/alcalase mass ratio; C, total hydrolysis time.
